# An advanced network pharmacology study to explore the novel molecular mechanism of Compound Kushen Injection for treating hepatocellular carcinoma by bioinformatics and experimental verification

**DOI:** 10.1186/s12906-022-03530-3

**Published:** 2022-03-02

**Authors:** Shan Lu, Ziqi Meng, Yingying Tan, Chao Wu, Zhihong Huang, Jiaqi Huang, Changgeng Fu, Antony Stalin, Siyu Guo, Xinkui Liu, Leiming You, Xiaojiaoyang Li, Jingyuan Zhang, Wei Zhou, Xiaomeng Zhang, Miaomiao Wang, Jiarui Wu

**Affiliations:** 1 Department of Clinical Chinese Pharmacy, School of Chinese Materia Medica, Beijing University of Chinese Medicine, No. 11 of North Three-ring East Road, Chao Yang District, Beijing, 102488 China; 2 Xiyuan Hospital of China Academy of Chinese Medical Sciences, Beijing, 100091 China; 3 Institute of Fundamental and Frontier Sciences, University of Electronic Science and Technology of China, Chengdu, 610054 China; 4 Department of Immunology and Microbiology, School of Life Science, Beijing University of Chinese Medicine, Fangshan District, Beijing, 102488 China; 5 China-Japan Friendship Hospital, Beijing, 100029 China

**Keywords:** Compound Kushen Injection, Hepatocellular carcinoma, Network pharmacology, Proliferation

## Abstract

**Background:**

Compound Kushen Injection (CKI) is a Chinese patent drug that exerts curative effects in the clinical treatment of hepatocellular carcinoma (HCC). This study aimed to explore the targets and potential pharmacological mechanisms of CKI in the treatment of HCC.

**Methods:**

In this study, network pharmacology was used in combination with molecular biology experiments to predict and verify the molecular mechanism of CKI in the treatment of HCC. The constituents of CKI were identified by UHPLC-MS/MS and literature search. The targets corresponding to these compounds and the targets related to HCC were collected based on public databases. To screen out the potential hub targets of CKI in the treatment of HCC, a compound-HCC target network was constructed. The underlying pharmacological mechanism was explored through the subsequent enrichment analysis. Interactive Gene Expression Profiling Analysis and Kaplan-Meier plotter were used to examine the expression and prognostic value of hub genes. Furthermore, the effects of CKI on HCC were verified through molecular docking simulations and cell experiments in vitro.

**Results:**

Network analysis revealed that BCHE, SRD5A2, EPHX2, ADH1C, ADH1A and CDK1 were the key targets of CKI in the treatment of HCC. Among them, only CDK1 was highly expressed in HCC tissues, while the other 5 targets were lowly expressed. Furthermore, the six hub genes were all closely related to the prognosis of HCC patients in survival analysis. Molecular docking revealed that there was an efficient binding potential between the constituents of CKI and BCHE. Experiments in vitro proved that CKI inhibited the proliferation of HepG2 cells and up-regulated SRD5A2 and ADH1A, while down-regulated CDK1 and EPHX2.

**Conclusions:**

This study revealed and verified the targets of CKI on HCC based on network pharmacology and experiments and provided a scientific reference for further mechanism research.

**Supplementary Information:**

The online version contains supplementary material available at 10.1186/s12906-022-03530-3.

## Background

Globally, the incidence of liver cancer is increasing annually, and accounts for a large proportion of cancer cases and deaths [1]. According to the statistics in 2020, liver cancer ranks seventh in malignant tumor incidence and third in mortality [2]. Only in China, both the incidence and mortality of liver cancer are higher than the global average, and the survival rate of liver cancer is very low, which is a heavy burden on society and medical care worldwide [3–6]. Primary liver cancer includes different pathological subtypes, such as hepatocellular carcinoma (HCC), intrahepatic cholangiocarcinoma (ICC), and mixed hepatocarcinoma. HCC accounts for 75–85% of primary liver cancers and is the most common type [2, 7]. Due to its insidious onset, most HCC patients are at an advanced stage at the time of diagnosis. Unfortunately, local (chemoembolization) and surgical treatments are relatively disappointing in improving overall survival (OS) of advanced stage patients. At the same time, traditional chemotherapy methods also do not show promising efficacy in treating HCC due to significant side effects [8–10]. Therefore, it is essential to explore new prognostic markers for HCC and develop more effective drugs with less toxic effects.

In recent decades, traditional Chinese medicine (TCM) has been shown to play an important role in the treatment of tumors by inhibiting tumor proliferation, reducing tumor recurrence and metastasis, prolonging survival, and reducing the side effects of conventional treatments, tumors, etc. [11–13]. In particular, Chinese medicine injections, which have unique advantages in the treatment of tumors, are widely used in clinical practice because of their characteristics of the convenient application and rapid efficacy without irritating the gastrointestinal tract, as is the case with oral TCM [14–16].

Compound Kushen Injection (CKI), a kind of antineoplastic injection commonly used in clinical practice [17–19], consists of Kushen (*Radix sophorae flavescentis*) and Baituling (*Rhizoma smilacis glabrae*). Early studies have shown that CKI has a significant inhibitory effect on human HCC cells such as SMMC-7721, Hepa1–6, and LPC-H12 [19–21]. In addition, CKI significantly improved the clinical symptoms and quality of life of patients with advanced HCC. The combination of CKI and transcatheter arterial chemoembolization (TACE) has been reported to be effective in the treatment of advanced HCC and is therefore worthy of clinical application [22, 23]. The detailed information of CKI can be found in Supplementary file 1.

**Fig. 1:**
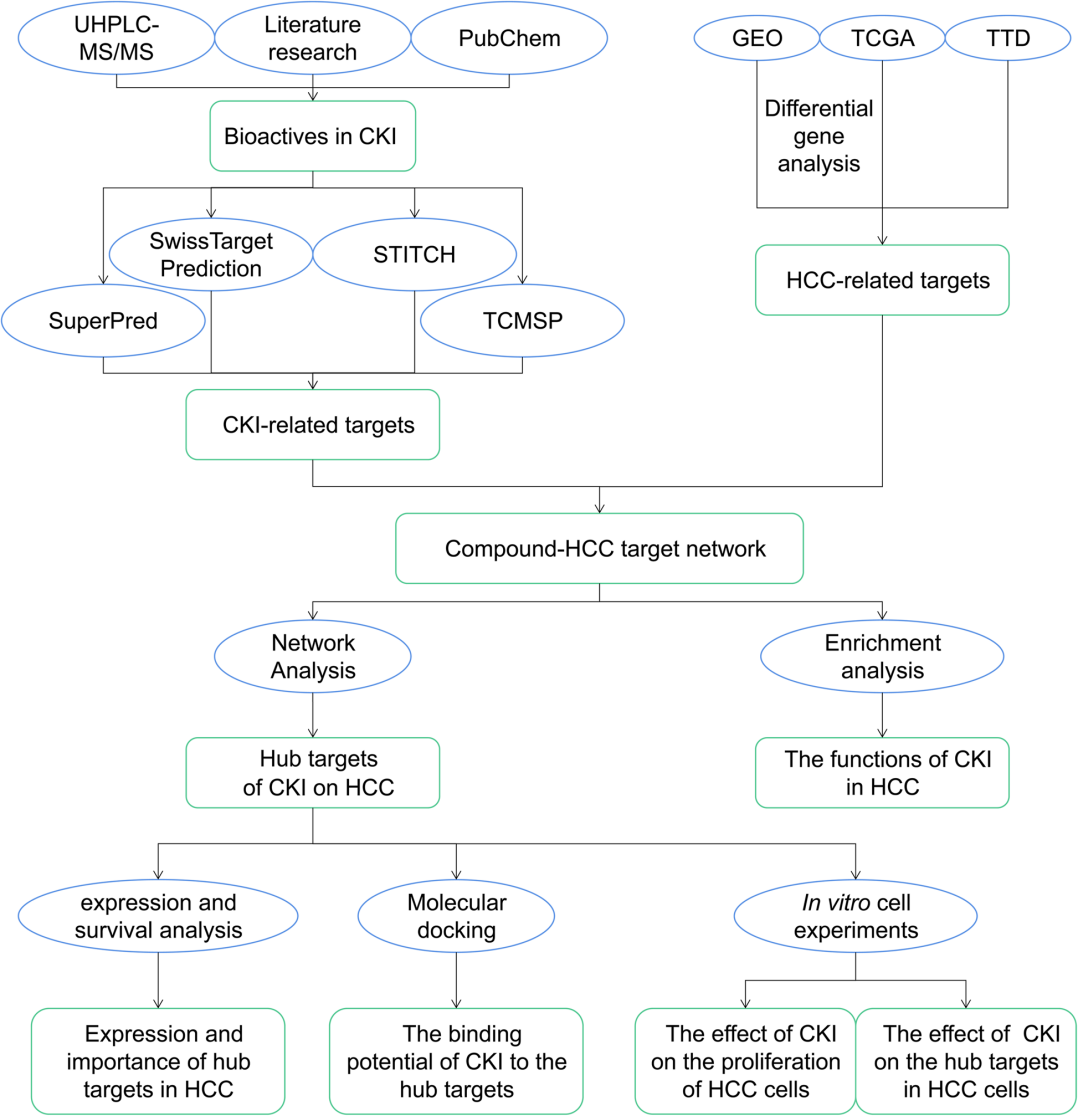
Workflow of exploring the potential pharmacological mechanism of CKI in the treatment of HCC

TCM preparations exert their special therapeutic effects by acting on the biological network in humans. Therefore, it is difficult to elucidate the specific mechanism of TCM in vivo [24, 25]. Until recently, network pharmacology provides a new method to promote the understanding of drug mechanisms [26, 27]. Network pharmacology constructs multicomponent and multitarget models to clarify better the complex interactions between genes, proteins, and metabolites related to diseases and drugs from a network perspective [28, 29]. In addition, molecular docking provides a relatively fast and economical alternative to standard experimental techniques that plays a vital role in the new drug development and discovery projects [30, 31]. AutoDock Vina software is often used to analyze the molecular interactions between protein and ligand [32]. To further explore and predict the molecular mechanism of CKI in the treatment of HCC and to identify targets related to the prognosis of HCC, network pharmacology was used in the present study. In addition, we further verified some of the potential targets of CKI through experiments in vitro. The flowchart of the current study is shown in Fig. 1.

## Materials and methods

### Identification of compounds and collection of potential targets for CKI

To identify the constituents of CKI, we conducted a qualitative analysis of CKI using UHPLC-MS/MS. Separations were performed on the Nexera LC-40 system (Shimadzu, Japan) using a Hypersil BDS (150 mm × 4. 6 mm, 5 μm). The mobile phase comprised 0.1% ammonia in water (A) and carbinol (B) at a flow rate of 0.5 ml/min and was eluted with gradient elution: 0–1 min (5–20% B), 1–30 min (20–80% B), 30–60 min (80–60% B). The column temperature was maintained at 25 °C and the injection volume was 5 μl. Analyses were performed with the QE-Orbitrap-MS (Thermo Fisher, USA) using an electrospray ionization (ESI) system. MS was operated in positive mode with the capillary temperature set at 350 °C. The spray voltage in negative mode was 3800 V, while the spray voltage in positive mode was 3200 V. The flow rates for the sheath gas and aux gas were 35arb and 15arb respectively. Three collision energies were used for MS2: low, medium and high. The positive ion mode was 30 eV, 40 eV, 50 eV, while the negative ion mode was 30 eV, 50 eV, 70 eV. The scan mode was set to full scan/ddMS2 and the scan range was 100–1200 Da. The full scan resolution was set to 70,000 FWHM while the resolution of MS2 was 17,500 FWHM. Finally, the retention time, MS fragmentation and UV spectra were used to identify the labeled reference compounds contained in the sample.

Next, we investigated the other compounds in CKI that had been reported in the literature to supplement our list of CKI-ingredients, and 23 compounds were selected for further study [20, 33, 34]. The canonical simplified molecular input line entry specification (SMILES) of 16 compounds were retrieved from the PubChem database [35] (https://pubchem.ncbi.nlm.nih.gov/) and exported. To search for the targets corresponding to these compounds, we imported the above data into the Search Tool for Interactions of Chemicals (STITCH) [36] (http://stitch.embl.de/), SuperPred [37] (http://prediction.charite.de/), SwissTargetPrediction [38] (http://www.swisstargetprediction.ch/), and Traditional Chinese Medicine Database and Analysis Platform (TCMSP) [39] (http://tcmspw.com/tcmsp.php).

### Collection of HCC-related targets

Therapeutic Target Database (TTD), Gene Expression Omnibus (GEO), and Cancer Genome Atlas (TCGA) were the main sources of HCC-related targets. In the TTD [40] (http://bidd.nus.edu.sg/group/ttd/ttd.asp), HCC related targets were identified by searching for “hepatocellular carcinoma”. For the dataset in GEO [41] (http://www.ncbi.nlm.nih.gov/GEO/), the differentially expressed genes (DEG) in each microarray were first screened using the limma package [42]. Then the RobustRankAggreg package [43] was used to integrate genes that were generally identified as differentially expressed in different datasets. All datasets used for the analysis met the following two criteria: (1) The samples used in the dataset were tissues obtained from human HCC and corresponding adjacent or normal tissues. (2) The data set containing at least 40 samples. Data from the TCGA-LIHC project [44] were obtained from UCSC Xena (https://xenabrowser.net/datapages/) and analyzed in R using the edgeR package [45].

### Network establishment

In the current study, three undirected networks were constructed: (1) a compound-putative target network, which included compounds of CKI and their corresponding targets; (2) a compound-HCC target network, which contained shared targets of CKI-ingredients and HCC; and (3) a drug-compound-target-pathway network. The above networks were visualized using Cytoscape 3.7.1 [46] (http://cytoscape.org/). Using the “Analyze Network” tool in Cytoscape3.7.1 software, the topological characteristics of each node in the HCC-related CKI compound-putative target network, namely Degree, Betweenness, and Closeness, were calculated to evaluate the importance of each node in the network.

### Enrichment analysis

To illustrate the role of putative targets of CKI in the treatment of HCC in biological processes and signaling pathways, a Gene Ontology (GO) enrichment analysis and a Kyoto Encyclopedia of Genes and Genomes (KEGG) pathway enrichment analysis were performed using the Database for Annotation, Visualization, and Integrated Discovery [47] (DAVID, https://david.ncifcrf.gov/). These analyses were performed for the targets in the compound-HCC target network, and the results were visualized by the ggplot2 package (http://docs.ggplot2.org/current).

### Survival analysis and correlation analysis of hub genes

Kaplan-Meier plotter (KM plotter, http://kmplot.com/analysis/), a database of clinical and gene expression data used to study the molecular basis of disease and identify biomarkers related to survival [48, 49]. The database contains disease-free survival and overall survival data based on the GEO, EGA (European Genome-Phenome Archive), and TCGA databases to calculate the hazard ratio (HR) with a 95% confidence interval and the *P* value of the log-rank test to assess the association between gene expression and survival [50].

Visualization of the results of expression level analysis and correlation analysis was carried out through Gene Expression Profiling Interactive Analysis [50] (GEPIA, http://GEPIA.cancerpku.cn/index.html), a web-based tool that allows the analysis of different tumor data in TCGA and Genotype Tissue Expression (GTEx). It contains 9736 tumor samples and 8587 normal tissue samples from 33 types of malignant tumors [51]. In addition, GEPIA provides customizable features such as differential expression analysis of tumors and normal. Using GEPIA, we have demonstrated the expression of key targets in HCC and normal tissues and the correlation between these targets.

### Molecular docking

The molecular docking includes the following three steps:

1. The preparation of the receptors. The three-dimensional (3D) crystal structures of the key targets were extracted from the Research Collaboratory for Structural Bioinformatics (RCSB) Protein Database [52] (PDB, https://www. rcsb.org/), and the protein structures of these key targets were processed using AutoDock Tools (ADT) [53], including dehydration, hydrogenation, removal of small molecule ligands, and calculation of Gasteiger charge. The position of the active pocket was determined by the ligand in the crystal structure, and the structures were saved in *pdbqt format.
2. The preparation of ligands. The *SDF files of the 2D structure of all bioactives were downloaded from PubChem database (https://pubchem.ncbi.nlm.nih.gov/). Next, they were converted into the corresponding 3D structures with the help of ChemOffice software (https://www.chemdraw.com.cn/product.html), and their energy were minimized and saved as *mol2 files. Finally, the small ligand molecules in *mol2 format were exported to *pdbqt format through ADT.
3. Molecular docking simulation. Through AutoDock Vina [54], the molecular docking simulation of the bioactives and the target protein were carried out in turn, and the Affinity were extracted. The results were visualized and analyzed by PyMOL (http://www.PyMOL.org).

### In vitro cell experimental verification

#### Reagents and cell line

CKI (Batch No. 20200329, total alkaloid concentration of 25 mg/ml) was provided by Shanxi Zhendong Pharmaceutical Co., Ltd. (China). Dulbecco modified eagle medium (DMEM), fetal bovine serum (FBS), penicillin-streptomycin, trypsin-EDTA, and phosphate buffered saline (PBS) were purchased from GIBCO (NY, USA). Cell Counting Kit-8 (CCK8) and BeyoClick™ EdU Cell Proliferation Kit with Alexa Fluor 488 were purchased from Beyotime (Beijing, China). RIPA lysis buffer, phenylmethylsulfonyl fluoride (PMSF) and BCA Protein Assay Kit were obtained from Biorigin (Beijing, China). Primary antibodies steroid 5-α-reductase 2 (SRD5A2) were purchased from Bioss (Beijing, China). Alcohol dehydrogenase 1A (ADH1A) was purchased from Solarbio (Beijing, China). Soluble epoxide hydrolase 2 (EPHX2), cyclin-dependent kinase 1 (CDK1), β-actin, and secondary antibody (anti-rabbit IgG and anti-mouse IgG) were procured from Proteintech (Wuhan, China). Nitrocellulose (NC) membranes and Immobilion Western Chemilum HRP Substrate were purchased from Merck Millipore (USA). Trizol reagent was obtained from Invitrogen (USA). SYBR Green Realtime PCR Master Mix was purchased from TOYOBO (Japan).

The human hepatocellular carcinoma cell line HepG2 was obtained from Procell (Wuhan, China) and cultured in DMEM supplemented with 10% FBS and 1% penicillin-streptomycin. Cells were maintained in a cell incubator at 37 °C and 5% CO_2_.

#### Cell proliferation assays

HepG2 cells in the logarithmic growth phase were processed, into a cell suspension, and seeded in 96-well plates at a density of 1 × 10^4^ cells / well. After 24 h, cells were treated with different concentrations of CKI (0, 0.125, 0.25, 0.5, 1, 2, 4, 8, and 16 mg/ml) diluted with DMEM for 24 h, 48 h, and 72 h, respectively. Then, 10 μl of CCK8 reagent was added to each well to detect cell proliferation. The viability of the CKI treated cells was calculated using the optical density (OD) at 450 nm.

$$\mathrm{Cell}\ \mathrm{viability}=\left({\mathrm{OD}}_{\mathrm{CKI}}-{\mathrm{OD}}_{\mathrm{blank}}\right)/\left({\mathrm{OD}}_{\mathrm{control}}-{\mathrm{OD}}_{\mathrm{blank}}\right)\times 100\%$$

The EdU staining method was used to verify the effect of CKI on the proliferation of HepG2 cells. Cells were seeded in a confocal dish and treated with different concentrations of CKI (0, 1, 2, 4 mg/ml) after 24 h. According to the instructions of the EdU kit, the HepG2 cells treated with CKI for 48 h were fluorescently stained. Then the cells were observed with a fluorescence microscope (Olympus FV3000, Japan).

#### Real-time polymerase chain reaction (RT-PCR)

The Trizol reagent was used to extract total RNA from the cell samples under the same treatment conditions as in the WB experiment. The SYBR Green Realtime PCR Master Mix was used to determine mRNA expression in each sample according to the manufacturer’s protocols. Then the 2^-ΔΔCT^ method was applied to calculate the relative expression level of the target gene. The primers used in the PCR were synthesized by Shenggong Biotech (Table 1).

**Table 1:** Primer sequences for RT-PCR

Genes	Primers	
GAPDH	Forward	TGGAGTCCACTGGCGTCTTCAC
	Reverse	TTGCTGATGATCTTGAGGCTGTTGTC
ADH1A	Forward	AAAACCCGGAGAGCAACTAC
	Reverse	CCACAGCCAATGAGACAGAC
CDK1	Forward	AAACTACAGGTCAAGTGGTAGCC
	Reverse	TCCTGCATAAGCACATCCTGA
EPHX2	Forward	ACCGAAACATGGAAAGGAA
	Reverse	GGGACATCTGAGGAACGAG
SRD5A2	Forward	GCAGTGTCTTAGTTGATGAG
	Reverse	TGTGGTTATTAAAACCTGGC

#### Western blot (WB)

HepG2 cells treated with CKI (0, 1, 2, 4 mg/ml) for 48 h were collected and total protein was extracted using RIPA lysis buffer supplement with 1% PMSF. The BCA method was used to calculate the protein concentration. After that, 5 × loading buffer was added to the protein samples and heated at 99 °C for 15 min to denature the protein. Then, the protein samples (20 μg/lane) were separated by 10% SDS-PAGE gel electrophoresis and transferred to NC membranes. Subsequently, the membranes were blocked in 5% skim milk for 1 h at room temperature. Afterward, the specific primary antibodies were added and incubated overnight at 4 °C, followed by incubation with the secondary antibodies at room temperature for 1 h. Finally, Immobilion Western Chemilum HRP Substrate was used to visualize the protein bands, and the gray values of the bands were quantified by ImageJ software (https://imagej.nih.gov/ij/).

#### Statistical analysis

Statistical analysis of experimental data was performed by GraphPad Prism8 (Inc. La Jolla, CA, United States). All data were expressed as mean ± standard deviation (SD) and were evaluated by Dunnett’s multiple comparisons test of one-way ANOVA. *P*-values of < 0.05 were considered to indicate a statistically significant difference. The calculation of the topological characteristics (Degree, Betweenness and Closeness) of each node in the network was performed using the “Analyze Network” tool in Cytoscape3.7.1.

## Results

### Construction of compound-HCC target network

**Fig. 2:**
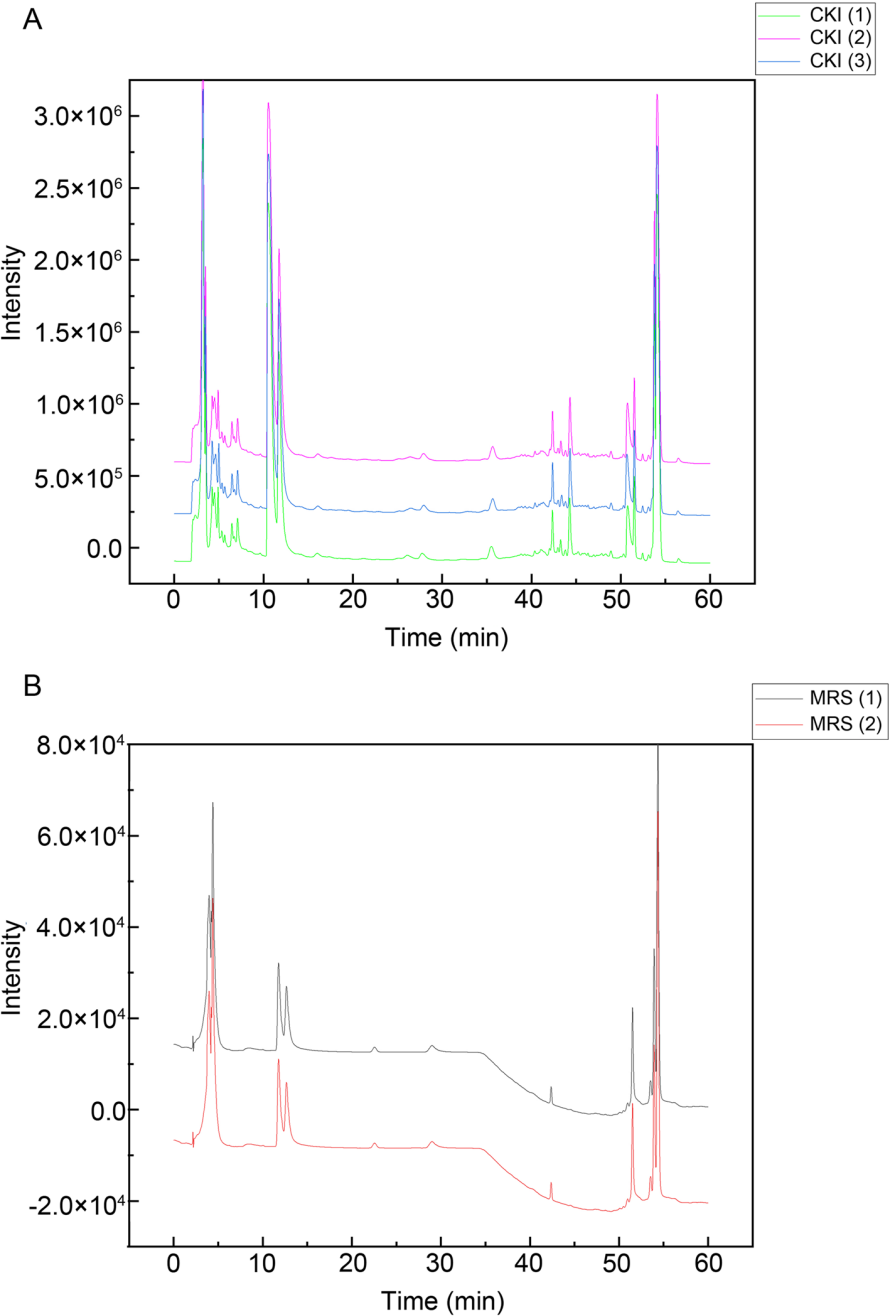
The result of UHPLC-MS/MS. (A) CKI. (B) Mixed reference standards (MRS)

**Fig. 3:**
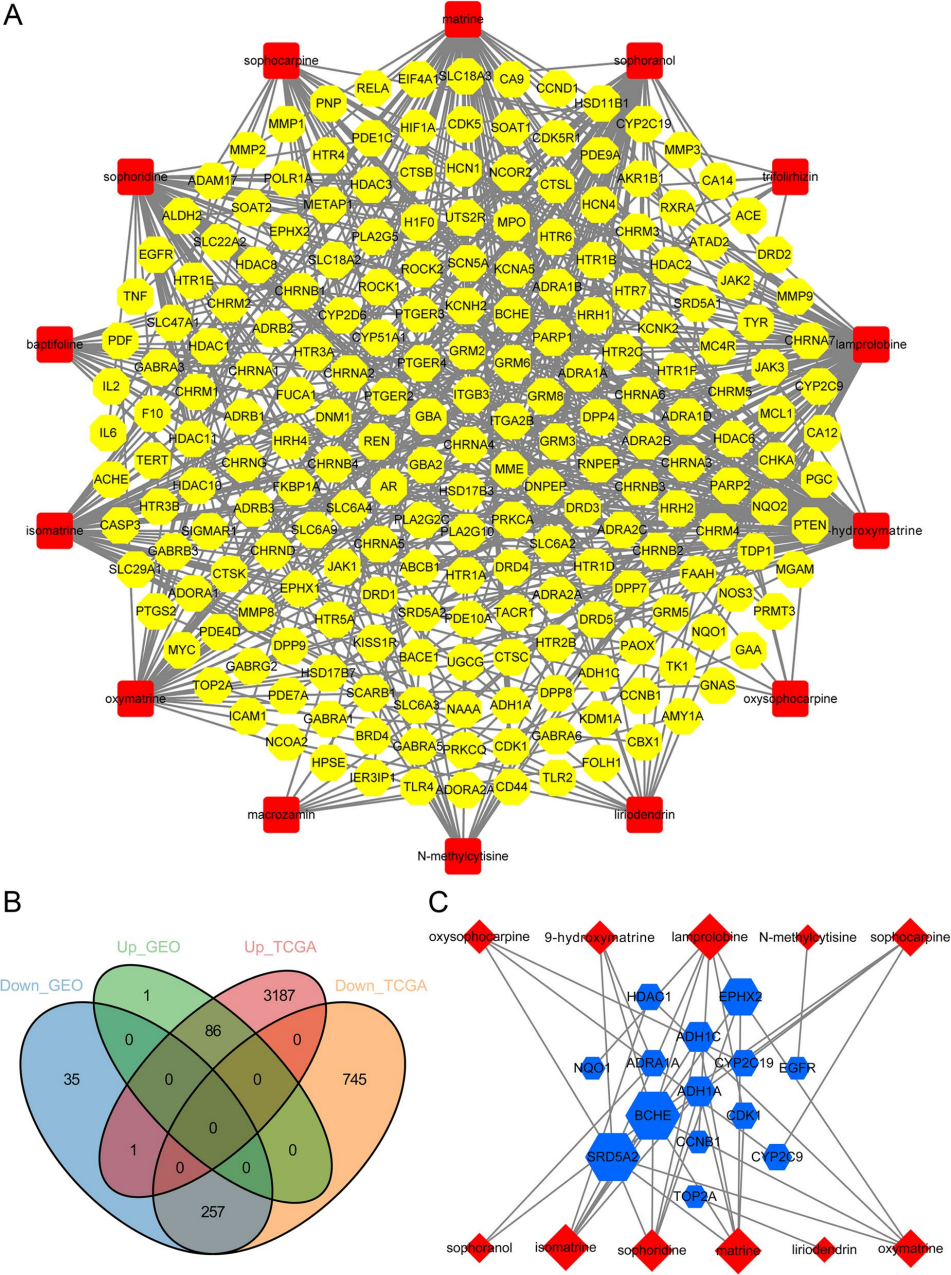
Construction of compound-HCC target network. **A** Compound-putative target network of CKI (red round rectangles represent compounds, and yellow octagons represent targets). **B** Identification of DEGs in TCGA and GEO. **C** Compound-HCC target network (red diamonds represent compounds, and blue hexagons represent targets of CKI for HCC treatment, the size of the node was proportional to its degree)

Through UHPLC-MS/MS, 10 compounds were identified in CKI (Table 2). Through a literature search, additional 13 compounds were added in CKI. Finally, the 3D-chemical structures of 14 chemical components and 217 corresponding targets were obtained from public databases such as PubChem, STITCH, SuperPred and TCMSP. Table 2 shows the basic information of the 14 constituents in CKI. As shown in Fig. 2, the distributions of the mixed reference standards and the samples in the chromatogram were approximately the same. Based on the above data, the compound-putative target network (Fig. 3A) was established, including 231 nodes (14 compound nodes and 217 target nodes) and 555 edges.

**Table 2:**
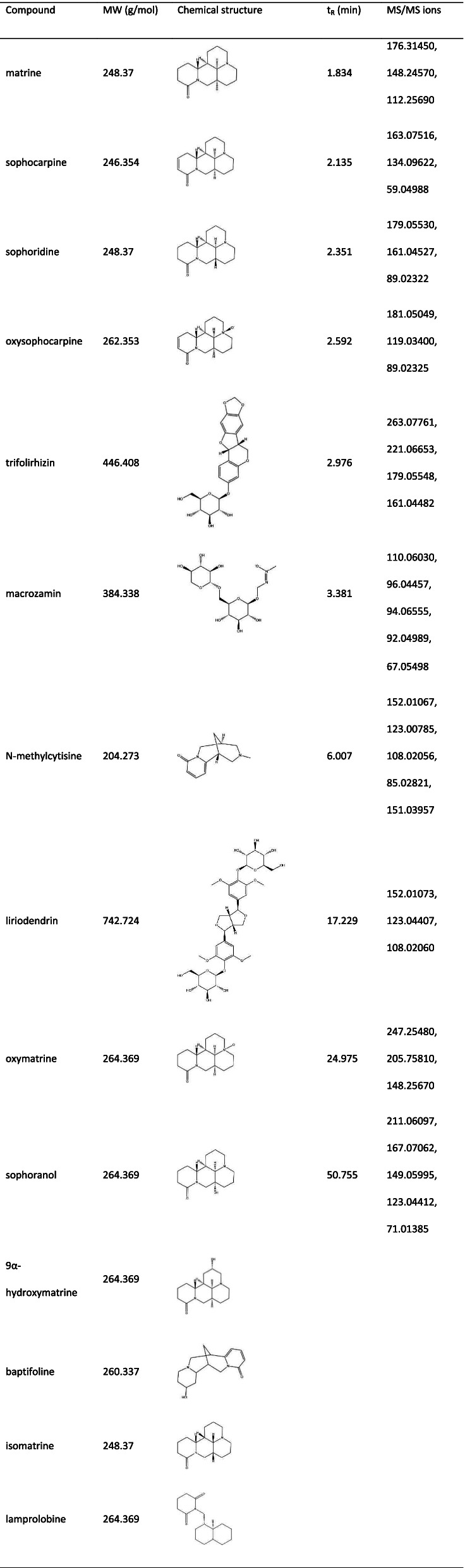
Basic information of the 14 compounds in CKI

By consulting TTD, GEO, and TCGA, we identified HCC-related targets, of which 380 DEGs were obtained from GEO and 4276 DEGs were obtained from TCGA. After taking the intersection of the above two DEG sets, 343 DEGs were obtained (Fig. 3B). Integrating the 16 targets related to the treatment of HCC from TTD and the DEGs as above-mentioned, a total of 358 disease targets were obtained. Detailed information on the 358 disease targets that can be found in Supplementary file 2.

In order to explore the targets of CKI in the treatment of HCC, the CKI compound-putative target network and the targets related to HCC were merged, and the targets that did not overlap were removed. Then, 14 putative targets of CKI in the treatment of HCC were intuitively identified. As shown in Fig. 3C, the HCC-related CKI compound-putative target network consisted of 25 nodes (11 compound nodes and 14 target nodes) and 41 edges.

### GO and KEGG enrichment analysis

**Fig. 4:**
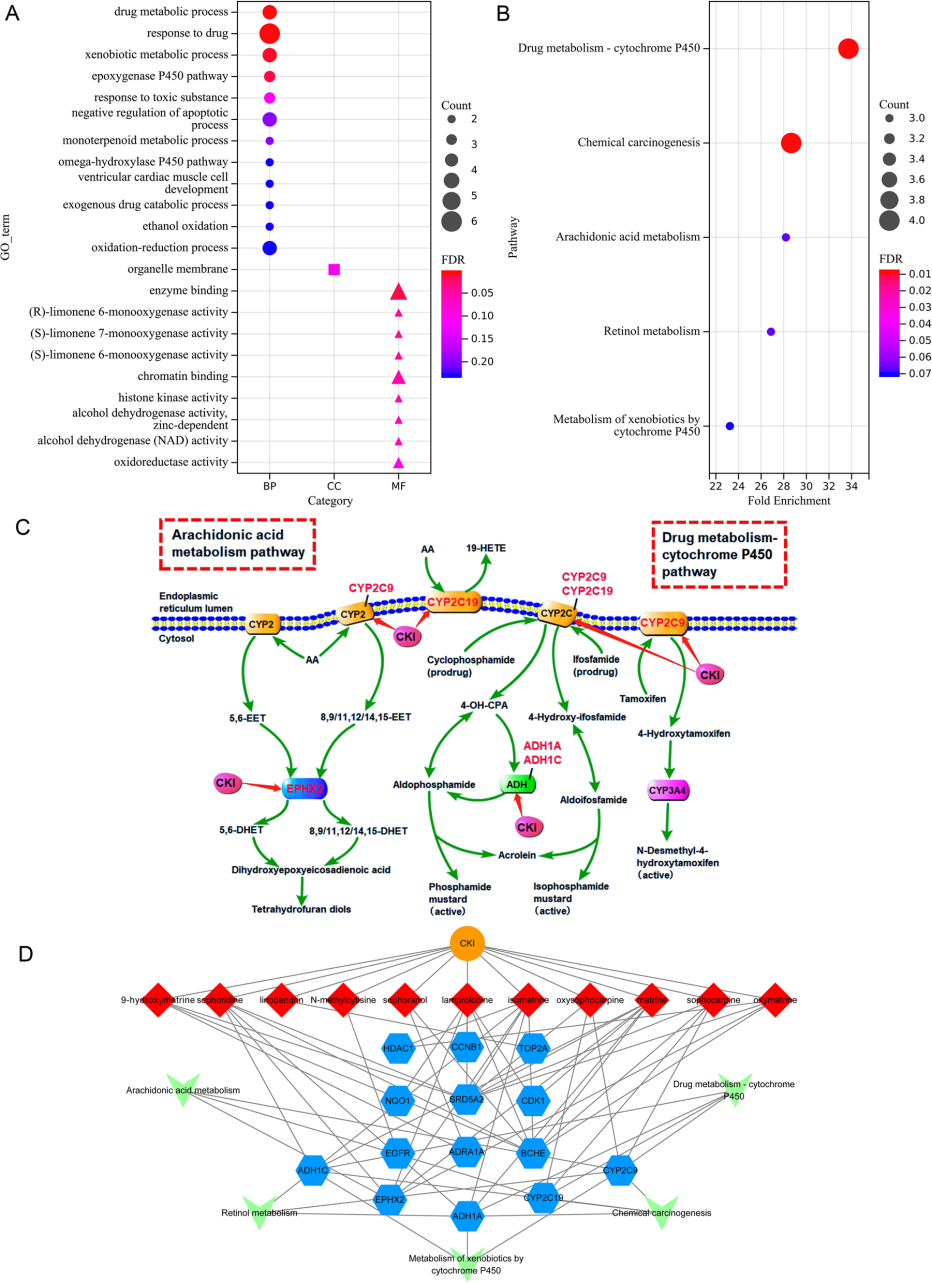
Enrichment analysis of the compound-HCC target network. **A** The result of GO enrichment analysis. **B** The result of KEGG enrichment analysis. **C** Arachidonic acid metabolism pathway and the drug metabolism-cytochrome P450 pathway. **D** Drug-compound-target-pathway network (orange ellipses represent CKI, red diamonds represent compounds, blue hexagons represent shared targets of CKI and HCC, and green Vs represent pathways)

To clarify the pharmacological mechanism of CKI in the treatment of HCC from the system level, GO and KEGG enrichment analyses were performed for the 14 key targets. In the results of GO analysis, 22 entries were selected based on *P* < 0.01, of which 12 were biological processes mainly related to drug metabolic process, drug response and xenobiotic metabolic process, etc., 9 were molecular functions mainly involving enzyme binding, chromatin binding and oxidoreductase activity, etc., and 1 was cell components, i.e. organelle membrane (Fig. 4A). KEGG enrichment analysis showed that the key genes were significantly enriched in 5 pathways, namely Drug metabolism-cytochrome P450 (hsa00982), Chemical carcinogenesis (hsa05204), Arachidonic acid metabolism (hsa00590), Retinol metabolism (hsa00830) and Metabolism of xenobiotics by cytochrome P450 (hsa00980) (Fig. 4B). Figure 4C shows two important metabolic pathways, namely drug metabolism-cytochrome P450 and arachidonic acid metabolism pathway.

To systematically and holistically explain the mechanism of CKI in the treatment of HCC, Cytoscape software was used to construct a drug-compound-target-pathway network. As shown in Fig. 4D, there were a total of 31 nodes (1 CKI node, 11 compound nodes, 14 target nodes, and 5 pathway nodes) and 69 edges.

### Survival analysis and correlation analysis of hub genes

**Fig. 5:**
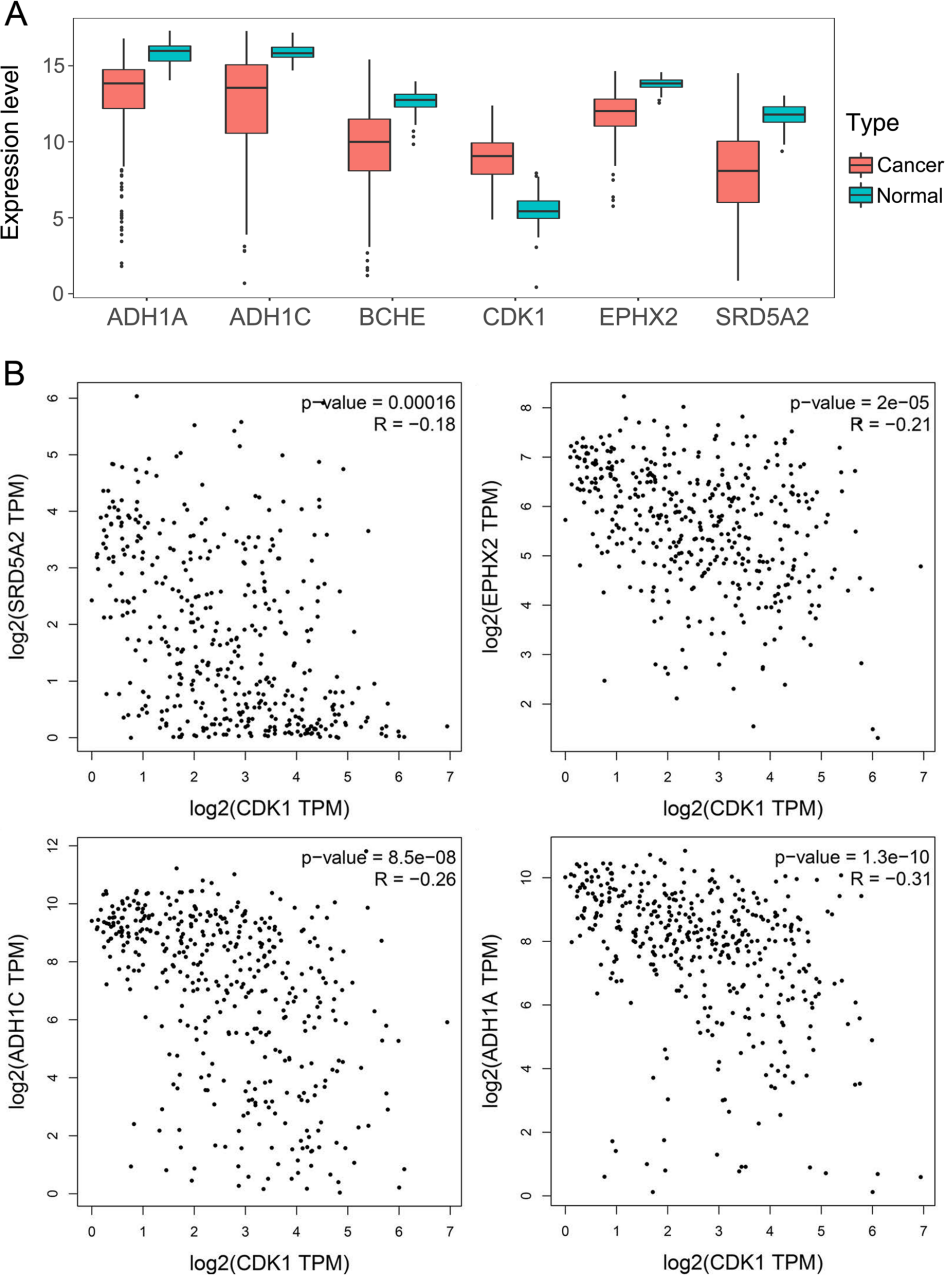
Expression and correlation analysis of the six hub genes of CKI in the treatment of HCC. **A** Expression levels of the six hub genes in the tumor and normal groups. **B** Correlation analysis between CDK1 and the other five important targets

According to the criterion of degree ≥2 (median degree), betweenness ≥0.0062 (average betweenness), closeness ≥0.3774 (average closeness), and edge count ≥2 (median edge count), six targets, including BCHE, SRD5A2, EPHX2, ADH1C, ADH1A, and CDK1, were identified as potential hub targets of CKI in the treatment of HCC. Figure 5A shows the expression levels of the six hub genes in tumor and normal tissues, respectively. BCHE, SRD5A2, EPHX2, ADH1C and ADH1A were lowly expressed in HCC, while CDK1 was highly expressed in HCC.

Correlation analysis was performed for CDK1 and the above 5 low-expressed genes (BCHE, SRD5A2, EPHX2, ADH1C, and ADH1A). As shown in Fig. 5B, the expression of SRD5A2, EPHX2, ADH1C, and ADH1A in HCC showed a strong negative correlation with the expression of CDK1.

**Fig. 6:**
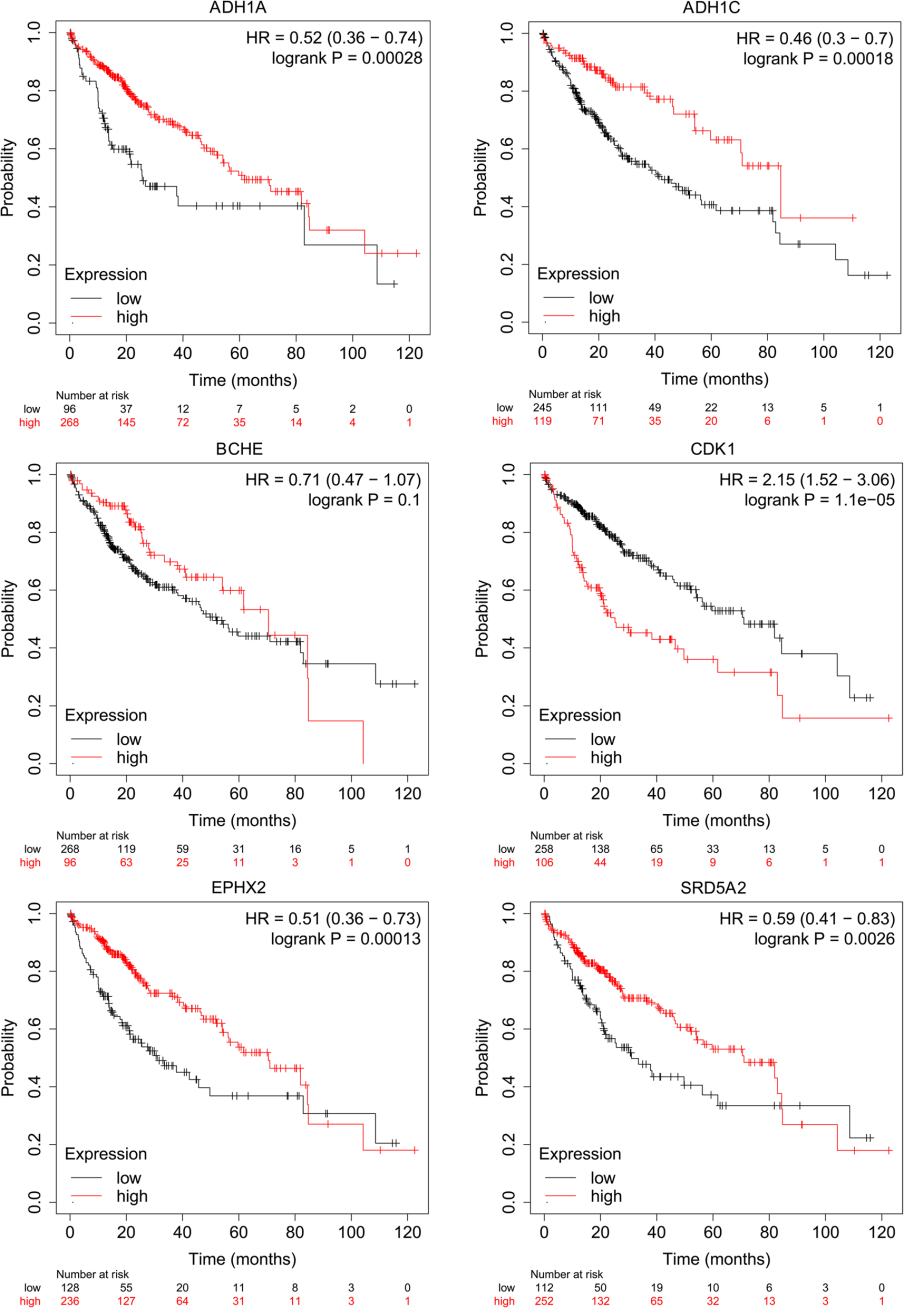
Survival analysis of the six hub genes of CKI in the treatment of HCC

Survival analysis of these six hub targets was performed by KM plotter. The results showed that low-expressed SRD5A2 [HR = 0.59 (0.41–0.83), *P* = 0.0026], EPHX2 [HR = 0.51 (0.36–0.73), *P* = 0.00013], ADH1C [HR = 0.46 (0.3–0.7), *P* = 0.00018], and ADH1A [HR = 0.52 (0.36–0.74), *P* = 0.00028] and high-expressed CDK1 were all related to a poor prognosis in HCC patients. Only BCHE was not independent of the OS of HCC (*P* > 0.05) (Fig. 6). However, the level of BCHE was found to be an important survival factor for patients with prostate cancer [55].

### Molecular docking verification

**Fig. 7:**
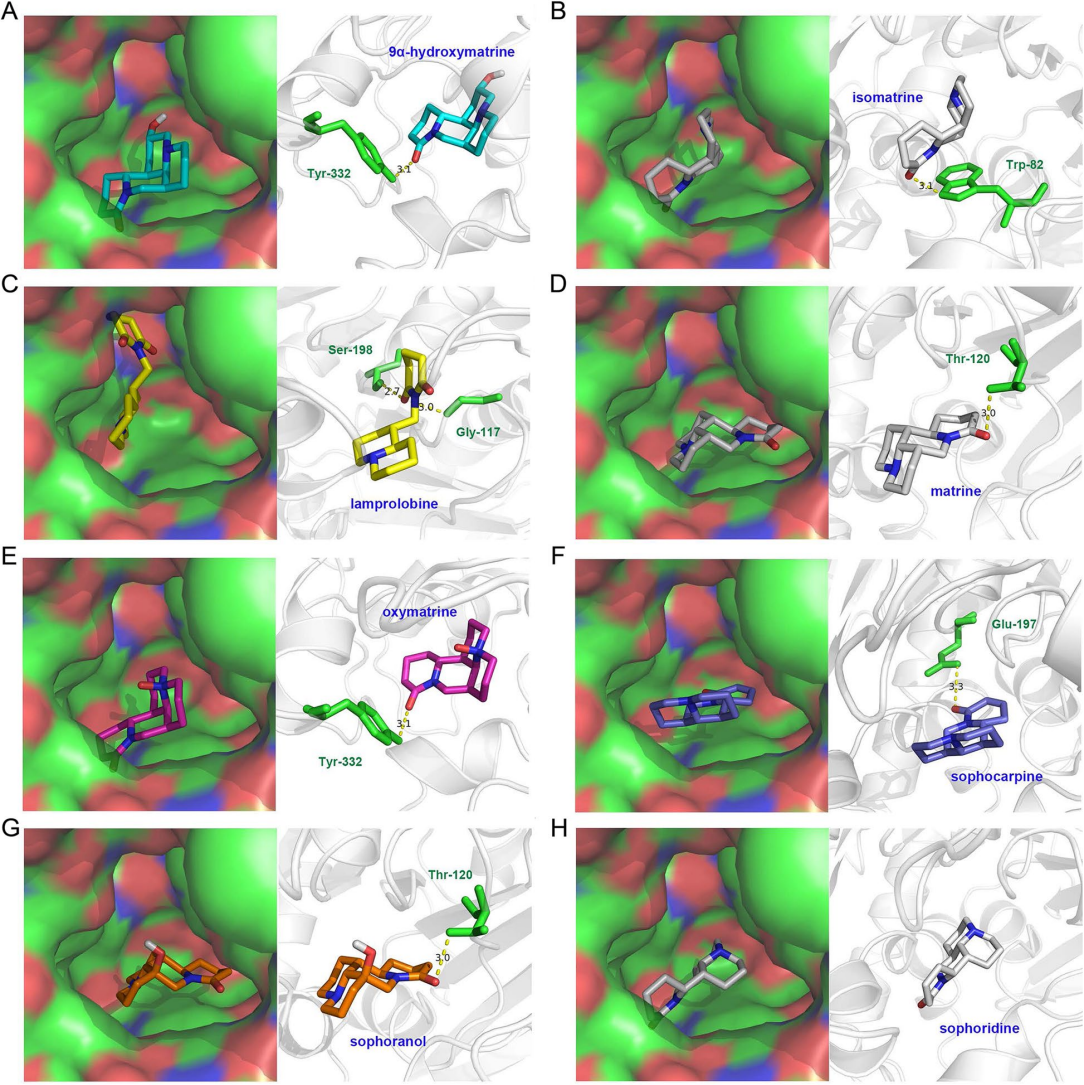
Detailed CKI ingredients-BCHE active site docking simulation

Molecular docking simulation was used to analyze the interaction between the eight active compounds of CKI (9α-hydroxymatrine, isomatrine, lamprolobine, matrine, oxymatrine, sophocarpine, sophoranol, and sophoridine) and BCHE, the target with the highest degree. The crystal structure of BCHE (PDB ID: 5K5E) was retrieved from the PDB, and the 3D structures of the eight compounds were downloaded from the PubChem database. The docking results are listed in Table 3. As shown in Fig. 7, 9α-hydroxymatrine formed a hydrogen bond with Tyr332; isomatrine formed a hydrogen bond with Trp82; lamprolobine formed hydrogen bonds with Ser198 and Gly117; matrine formed a hydrogen bond with Thr120; oxymatrine formed a hydrogen bond with Tyr332; sophocarpine formed a hydrogen bond with Glu197, and sophoranol formed a hydrogen bond with Thr120.

**Table 3:** Information on the docking of BCHE-related compounds with BCHE

Target	PDB ID	Compound	Affinity (kcal/mol)
BCHE	5K5E	sophoridine	−8.8
		hydroxymatrine	−8.5
		sophocarpine	−8.4
		matrine	−8.3
		oxymatrine	−8.3
		lamprolobine	−8.3
		isomatrine	−8.2
		sophoranol	−8.2

### In vitro cell experimental verification

#### CKI inhibits the proliferation of hepatocellular carcinoma cells

**Fig. 8:**
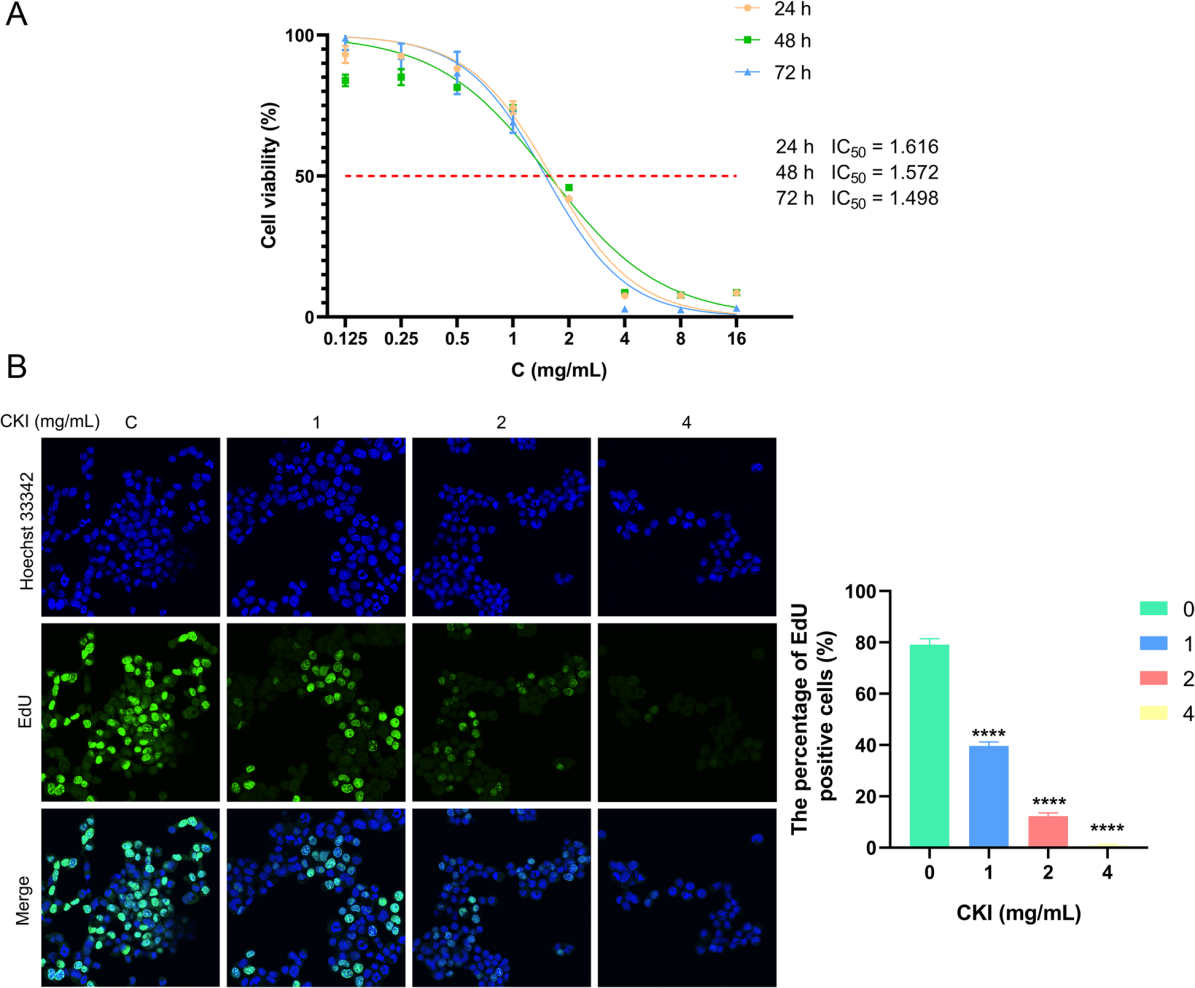
Effects of CKI on cell viability and proliferation of HepG2 cells. **A** Cell viability curve of HepG2 cells with CKI treated for 24 h, 48 h and 72 h. **B** The effect of CKI on the proliferation of HepG2 cells. Data are presented as mean ± SD of three independent experiments. (**** *P* < 0.0001)

To determine the effects of CKI on HepG2 cell viability, a CCK-8 assay was performed. As shown in Fig. 8A, CKI reduced the viability of HepG2 cells in a dose-dependent manner. In addition, the IC_50_ values of CKI after 24 h, 48 h, and 72 h were 1.616, 1.572 and 1.498 mg/ml, respectively. Also, the results of EdU showed f that the proliferation of HepG2 cells decreased with increasing dosage of CKI (Fig. 8B). Hoechst 33,342 staining showed that the number of HepG2 cells was significantly reduced under the intervention of 2 mg/ml and 4 mg/ml CKI for 48 h. Overall, these results suggest that CKI inhibits the growth and proliferation of HepG2 cells in a dose-dependent manner.

#### Regulations of CKI on ADH1A, CDK1, EPHX2, and SRD5A2

**Fig. 9:**
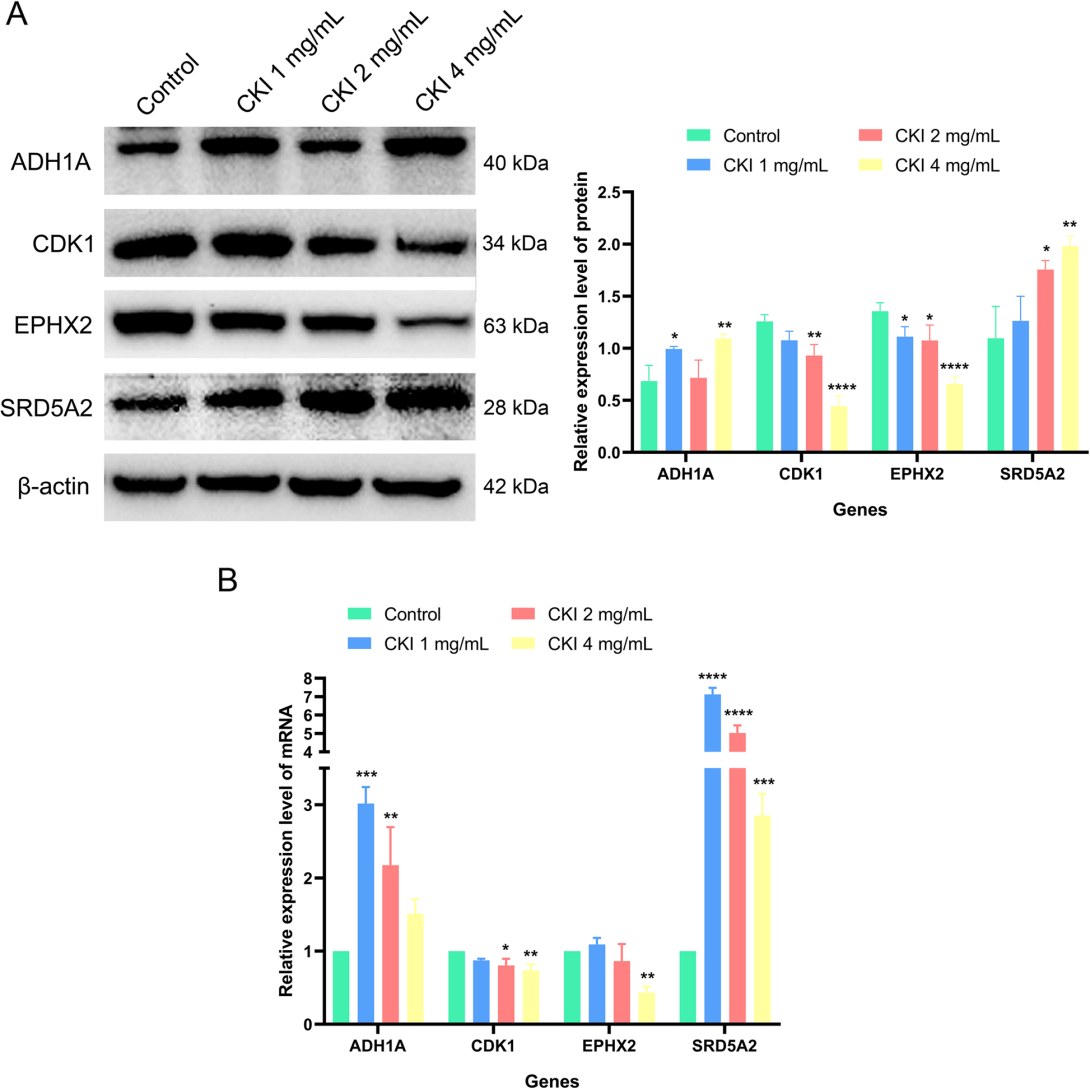
Effects of CKI on the expressions of ADH1A, CDK1, EPHX2 and SRD5A2 in HepG2 cells. **A** ADH1A, CDK1, EPHX2 and SRD5A2 protein in HepG2 cells with CKI treated. **B** ADH1A, CDK1, EPHX2 and SRD5A2 mRNA in HepG2 cells with CKI treated. Data are presented as mean ± SD of three independent experiments. (* *P* < 0.05; ** *P* < 0.01; *** *P* < 0.001; **** *P* < 0.0001)

The regulation of CKI on the potential targets (ADH1A, CDK1, EPHX2 and SRD5A2) in HepG2 cells were investigated by WB and RT-qPCR (Fig. 9A-B). The results showed that, CKI significantly increased the protein and mRNA expression of ADH1A and SRD5A2 in HepG2 cells compared with the control group. In contrast, the protein and mRNA expression of CDK1 and EPHX2 were significantly down-regulated by CKI. Notably, the prominent regulation of CKI on CDK1 was concentration-dependent.

## Discussion

HCC is the most common malignant tumor in the clinic and one of the leading causes of cancer-related deaths worldwide. In China, CKI is widely used in the treatment of cancer pain and is often combined with chemotherapy and radiotherapy in clinical practice [56]. Studies have shown that the combination of CKI with TACE has significant clinical efficacy in the treatment of HCC [22, 57]. In this study, the network pharmacology method was used in combination with molecular biology experiments to explore the potential targets and pharmacological mechanisms of CKI in the treatment of HCC.

Topological characteristics are important parameters for evaluating the importance of nodes in the network, including Degree, Betweenness and Closeness. “Degree” is defined as the number of edges associated with node *i*. “Betweenness” means the number of shortest paths between pairs of nodes passing through node *i*. “Closeness” represents the reciprocal of the sum of the distances between node *i* and the other nodes. The higher the above three node values, the greater the importance of the node in the network [58–61]. Based on the topological characteristics and edge counts of each node, we screened out 6 potential key targets (BCHE, SRD5A2, EPHX2, ADH1C, ADH1A, and CDK1) for CKI in the treatment of HCC. Among them, CDK1 was the only one highly expressed in HCC, and there was a strong negative correlation between its expression and the expression of the other five targets (BCHE, SRD5A2, EPHX2, ADH1C, and ADH1A).

Furthermore, the survival analysis results showed that high expression of CDK1 was closely related to the poor prognosis of HCC patients. In contrast, the high expression of SRD5A2, EPHX2, ADH1C and ADH1A indicated that HCC patients had a longer overall survival time. In addition, this study proved that CKI had a significant inhibitory effect on the proliferation of HepG2 cells.

Cyclin-dependent kinase 1 (CDK1) is a serine/threonine protein kinase and belongs to the cyclin-dependent kinase family (CDKs). The activated CDKs are necessary for cancer cell proliferation [62], and CDK1 is essential for the phase transition of G1/S and G2/M [63]. Zhao et al. found that CDK1 interacts with apoptin in HCC and is one of the key players in the activation of apoptin-induced tumor-specific apoptosis [64]. Clinical studies have shown that anti-CDK1 in HCC was one of the most effective ways to improve clinical efficacy [65]. In this study, we found that CKI caused a dose-dependent down-regulation of CDK1 in HepG2 cells. Mechanism studies have shown that matrine, one of the main ingredients of CKI, exhibits time-dependent inhibition of CDK1 expression in prostate cancer cells, resulting in o cell cycle arrest in the G0/G1 phase, thereby inhibiting cancer cell proliferation [66].

The alcohol dehydrogenase (ADH) family includes seven enzymes (ADH1–7) [67]. ADH1, including ADH1A, ADH1B, and ADH1C, are mainly expressed in the liver and play an important role in the conversion of ethanol to acetaldehyde, a kind of carcinogenic metabolite, especially in the excretory phase. Furthermore, these enzymes are essential for oral alcohol metabolism [68, 69]. Studies have shown that ADH1C polymorphisms were associated with various cancer risks such as gastric cancer and oral squamous cell carcinoma [70, 71]. It has been confirmed that low expression of ADH1C is associated with poor prognosis in patients with non-small cell lung cancer [67]. In HCC, both ADH1C and ADH1A have been shown to be downregulated [72, 73]. Inhibition of ADH1A expression would promote the occurrence and progression of HCC. However, high expression of ADH1A was related to a reasonable survival rate of HCC patients [74, 75]. Our experimental results showed that CKI increased the expression of ADH1A in HepG2 cells.

Butyrylcholinesterase (BCHE) is a glycoprotein synthesized by the liver and secreted into the bloodstream. It is a nonspecific cholinesterase and widely distributed in the nervous system, small intestine, and adipose tissue [76]. Recently, it has been reported to be a solid biochemical marker indicating liver organ damage. Functional destruction of the liver is associated with a decrease in plasma BCHE activity [77]. Therefore, BCHE has been found to be involved in the pathogenesis of chronic liver disease in patients with advanced HCC [78–81]. Besides, a lower level of BCHE in the serum is closely associated with the advanced stage and poor prognosis of various cancers, such as gastric cancer, renal cancer, bladder cancer, prostate cancer, and cervical cancer [76, 82–85]. Here, we carried out a molecular docking verification of the highest degree target BCHE and its related CKI-compounds. We found that all eight active compounds of CKI (9α-hydroxymatrine, isomatrine, lamprolobine, matrine, oxymatrine, sophocarpine, sophoranol, and sophoridine) had relatively high binding potential with the active site of BECE, indicating that there is likely an interaction between CKI and BCHE.

The EPHX2 gene encodes soluble epoxide hydrolase (sEH), a bifunctional enzyme of the epoxide hydrolase family, an enzyme that promotes increased apoptosis by inducing oxidative stress and inflammation [86]. Thomassen et al. found that sEH degrades biologically active epoxy fatty acids derived from arachidonic acid through the metabolism of P450 cytochromes (CYP) in the arachidonic acid pathway [87]. In addition, silencing of EPHX2 has been proved to reduce tumor cell viability, induce apoptosis, and inhibit androgen receptor signaling in prostate cancer [88]. For HCC, EPHX2 has been identified as a prognostic biomarker in previous studies [89]. In our research, CKI down-regulated the expression of EPHX2 in HCC.

SRD5A2 encodes steroid 5-α-reductase 2, an important enzyme in androgen metabolism. This gene has been found to expressed in cells sensitive to androgens, such as prostate, breast glands, and liver cells. It has been considered a risk factor for breast cancer and has been closely related to prostate cancer prognosis [90–93]. Previous studies have shown that SRD5A2 was down-regulated in tumors and negatively correlated with biochemical recurrence [91, 94]. In addition, epigenetic abnormal hypermethylation in the SRD5A2 promoter region can be used as an important molecular target for the detection of HCC [95], and the analysis of methylated SRD5A2 may help to accurately diagnose HCC, especially the early diagnosis of HCC [96, 97]. Our WB and PCR experimental results both showed that the expression level of SRD5A2 was significantly up-regulated in HepG2 cells after CKI treatment.

To further elucidate the multiple mechanisms of CKI in HCC from a systemic perspective, GO and KEGG enrichment analyses were performed. The results of GO analysis showed that CKI may be involved in drug response, metabolic drug process, and enzyme binding to affect HCC. In the metabolic pathway enrichment analysis, the potential targets of CKI were significantly enriched in drug metabolism-cytochrome P450, arachidonic acid metabolism and other metabolic pathways. Most of the drugs are metabolized in the liver by drug-metabolizing enzymes, and the primary drug-metabolizing enzymes are severely dysregulated in HCC, leading to chemotherapy failur e[98]. Moreover, dysfunction of the drug metabolism-cytochrome P450 pathway (hsa00982) has been reported to induce drug resistance or adverse reactions during chemotherapy in cancer by interrupting drug metabolism and promoting drug excretion [99]. Arachidonic acid metabolism has been proved to significantly impact the occurrence and development of various malignant diseases [100]. It has been reported that activation of arachidonic acid metabolism induces liver inflammation [101]. Studies have shown that berberine may induce apoptosis of HCC cells by inhibiting the metabolic pathway of arachidonic acid [102]. Therefore, we speculated that CKI may play an important therapeutic role in HCC by regulating drug metabolism-cytochrome P450 and arachidonic acid metabolic pathway.

## Conclusions

In this study, we systematically investigated the potential targets and pharmacological mechanisms of CKI in the treatment of HCC through network pharmacology analysis combined with enrichment analysis, survival analysis and other experimental methods. The network analysis showed that BCHE, SRD5A2, EPHX2, ADH1C, ADH1A and CDK1 were the key targets of CKI in treating HCC. The survival analysis results showed that SRD5A2, EPHX2, ADH1C, ADH1A and CDK1 were closely related to the prognosis of HCC patients. GO and KEGG enrichment analysis revealed that CKI could exert its therapeutic effect by regulating drug metabolism-cytochrome P450 and arachidonic acid metabolism. In addition, based on the cell experiments in vitro, we confirmed that CKI had a significant inhibitory effect on the proliferation of HepG2 cells. The results of WB and PCR experiments suggest that the anti-HCC effect of CKI may be related to the down-regulation of CDK1 and EPHX2 and the up-regulation of SRD5A2 and ADH1A. In future experiments, we would investigate the roles and functions of these key targets in more depth and details.

In conclusion, this study systematically analyzed the pharmacological mechanism of CKI in the treatment of HCC. Further, it confirmed the anti-cancer effects and potential targets of CKI in the clinical treatment of HCC.

## Supplementary Information

**Additional file 1: Table S1.** Detailed information of Compound Kushen injection.

**Additional file 2: Table S2.** Detailed information of 358 disease targets.

**Additional file 3: Figure S1.** Original images of WB.

## Data Availability

The datasets used and/or analyzed during the current study are available from the corresponding author on reasonable request.

## Abbreviations

ADH: Alcohol dehydrogenase
ADH1A: Alcohol dehydrogenase 1A
ADH1C: Alcohol dehydrogenase 1C
ADT: AutoDock Tools
BCHE: Butylcholinesterase
CDK1: Cyclin-dependent kinase 1
CDKs: Cyclin-dependent kinase
CCK8: Cell Counting Kit-8
CKI: Compound Kushen Injection
DAVID: Database for Annotation, Visualization, and Integrated Discovery
DEGs: Differentially expressed genes
DMEM: Dulbecco modified eagle medium
EGA: European Genome-Phenome Archive
EPHX2: Epoxide hydrolase 2
FBS: Fetal bovine serum
GEO: Gene Expression Omnibus
GEPIA: Gene Expression Profiling Interactive Analysis
GO: Gene Ontology
GTEx: Genotype Tissue Expression
HCC: Hepatocellular carcinoma
HR: Hazard ratio
ICC: Intrahepatic cholangiocarcinoma
KEGG: Kyoto Encyclopedia of Genes and Genomes
KM: Kaplan-Meier
NC membranes: Nitrocellulose membranes
OD: Optical density
OS: Overall survival
PBS: Phosphate buffered saline
PCR: Polymerase Chain Reaction
PDB: Protein Database
RCSB: Research Collaboratory for Structural Bioinformatics
SD: Standard deviation
sEH: Soluble epoxide hydrolase
SMILES: Simplified molecular input line entry specification
SRD5A2: Steroid 5-α-reductase 2
STITCH: Search Tool for Interactions of Chemicals
TACE: Transcatheter arterial chemoembolization
TCGA: The Cancer Genome Atlas
TCM: Traditional Chinese medicine
TCMSP: Traditional Chinese Medicine Database and Analysis Platform.
TTD: Therapeutic Target Database
WB: Western blot
