# Supplementary material for: An advanced network pharmacology study to explore the novel molecular mechanism of Compound Kushen Injection for treating hepatocellular carcinoma by bioinformatics and experimental verification
Source: BMC Complement Med Ther. 2022 Mar 2;22:54. doi: 10.1186/s12906-022-03530-3 (PMC8892752; doi:10.1186/s12906-022-03530-3)

**ADH1A**

1^st^ beta-actin_Chemiluminescence → beta-actin_brightfield → beta-actin_merge


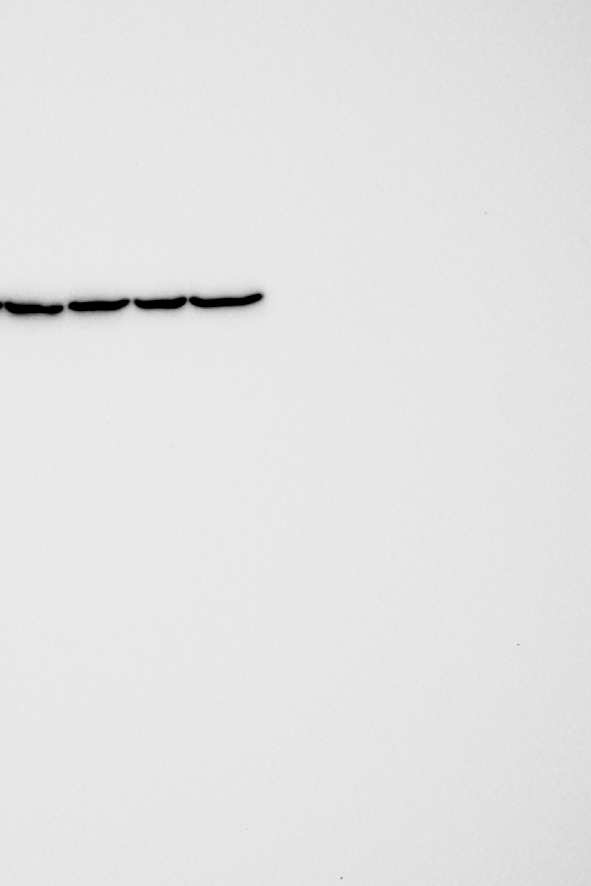

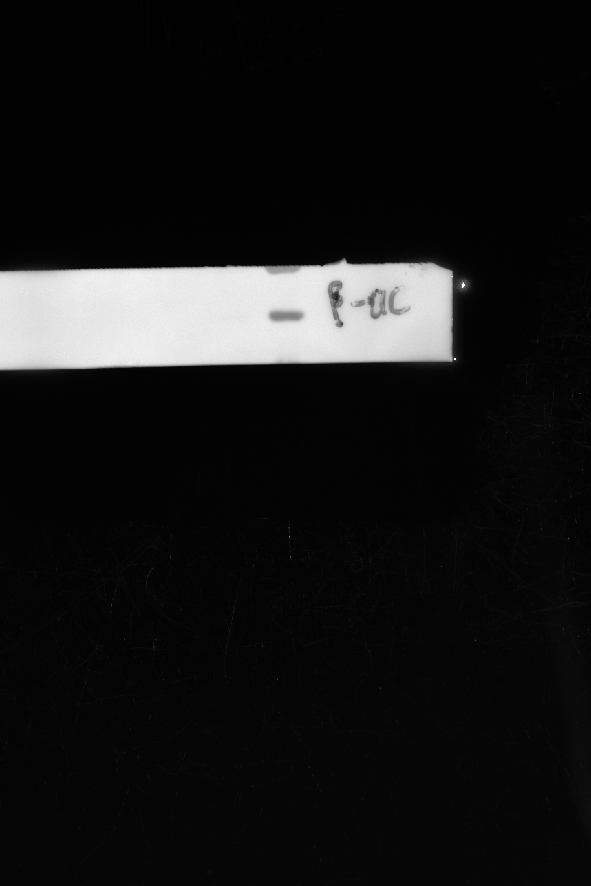

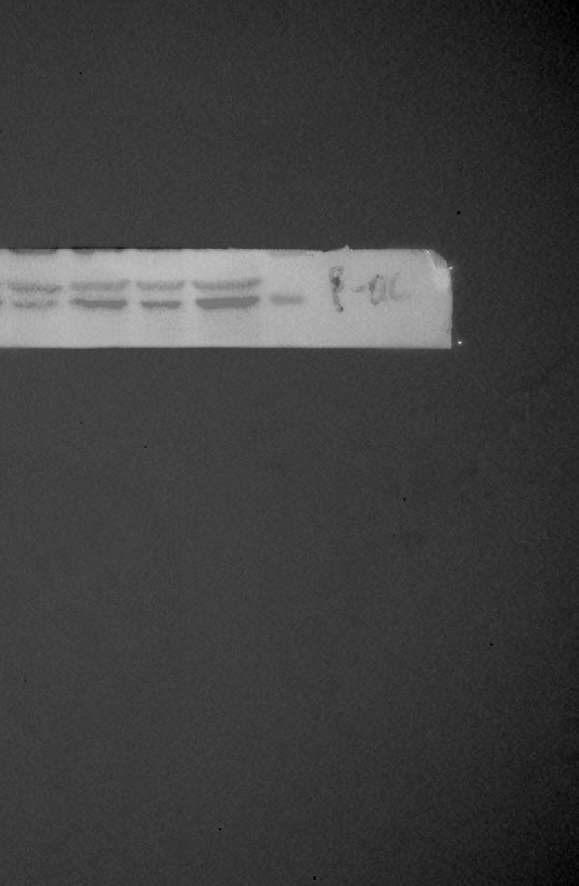


1^st^ ADH1A_Chemiluminescence → ADH1A_brightfield → ADH1A_merge → ADH1A_beta-actin_complication


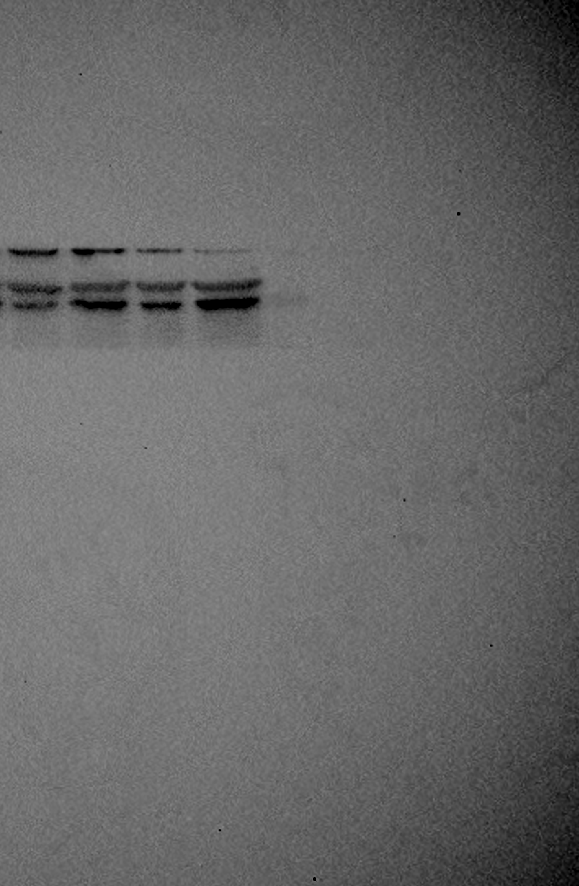

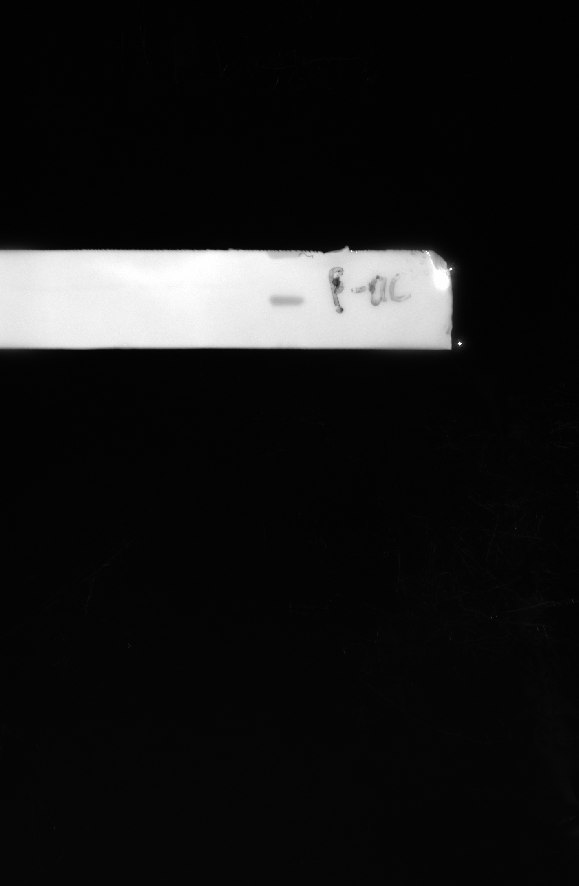

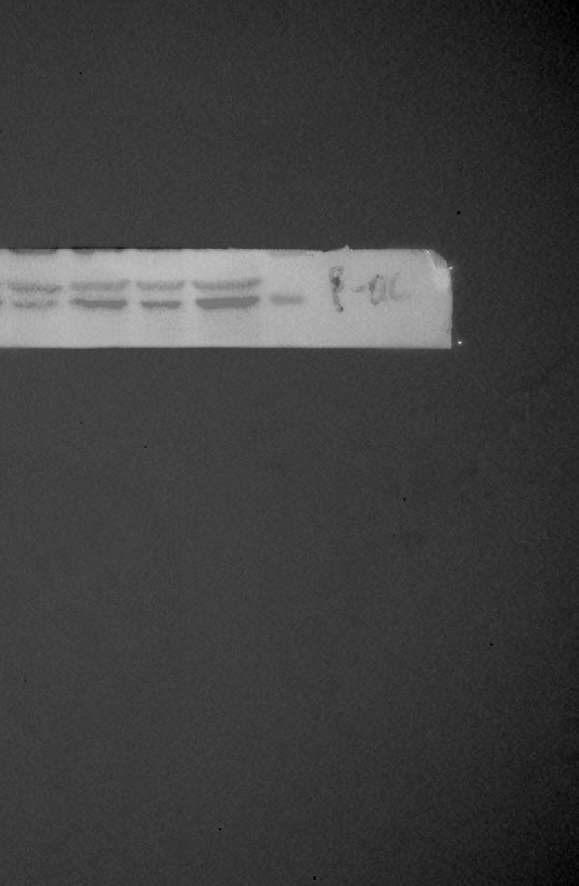

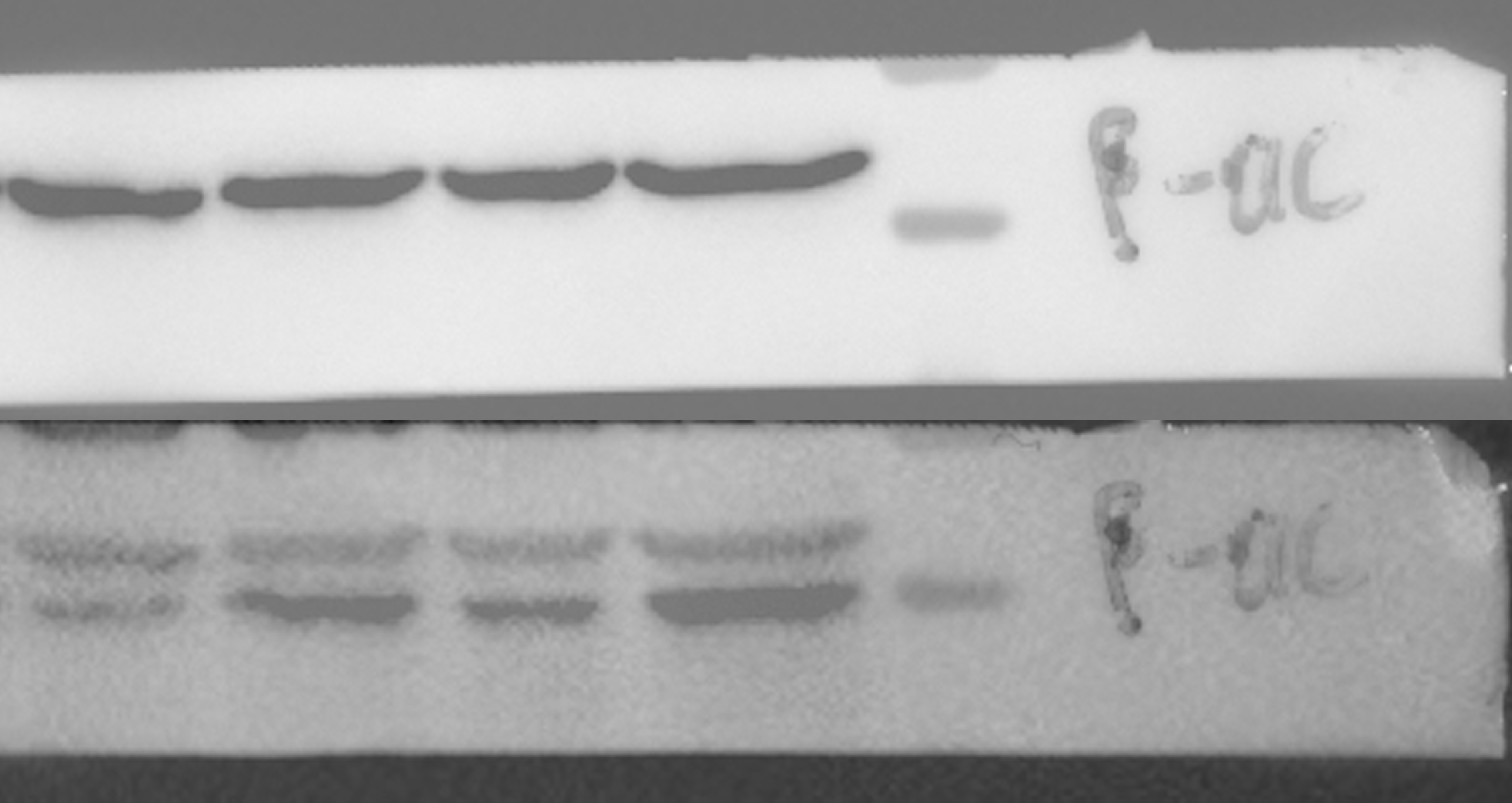


2^nd^ beta-actin_Chemiluminescence → beta-actin_brightfield → beta-actin_merge


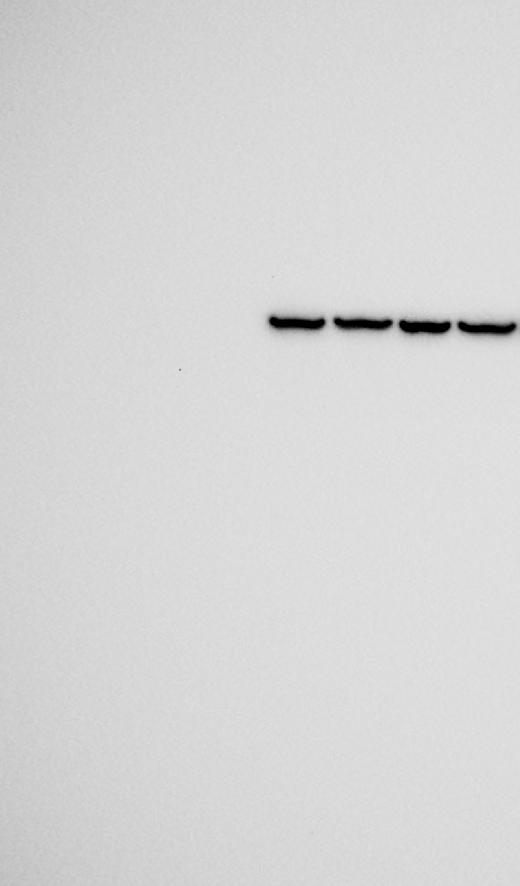

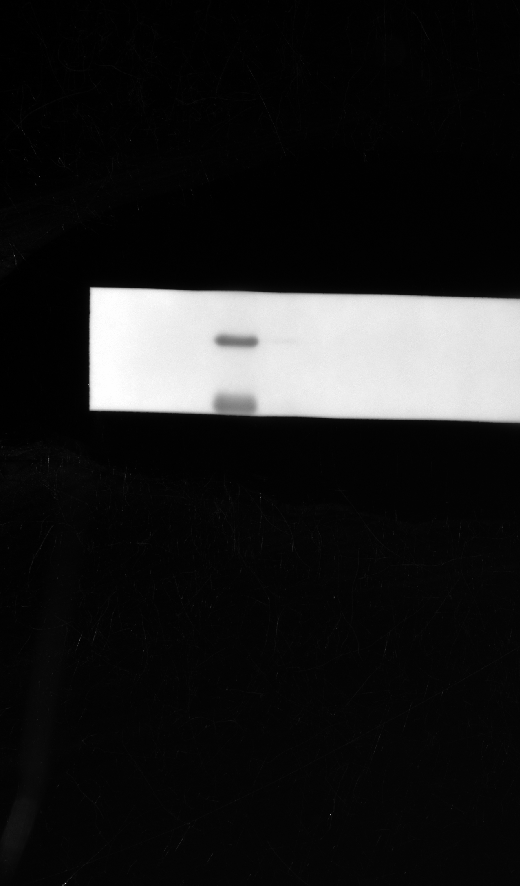

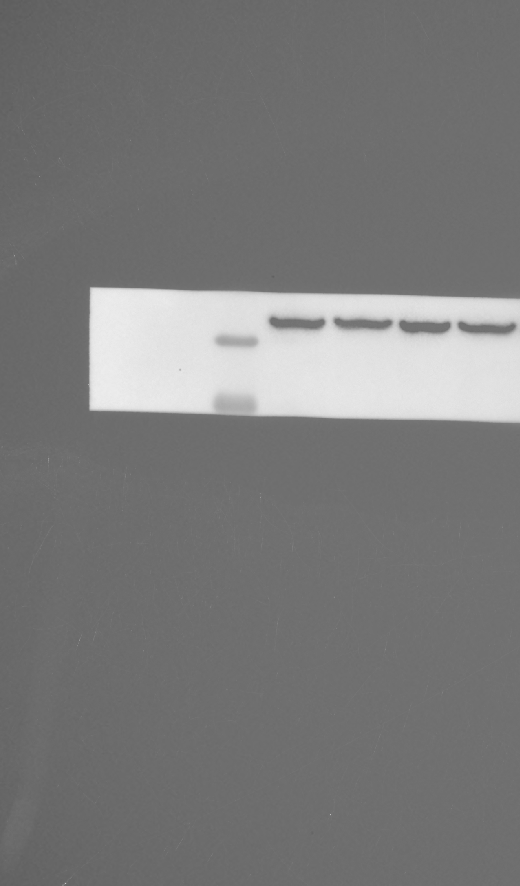


2^nd^ ADH1A_Chemiluminescence → ADH1A_brightfield → ADH1A_merge → ADH1A_beta-actin_complication


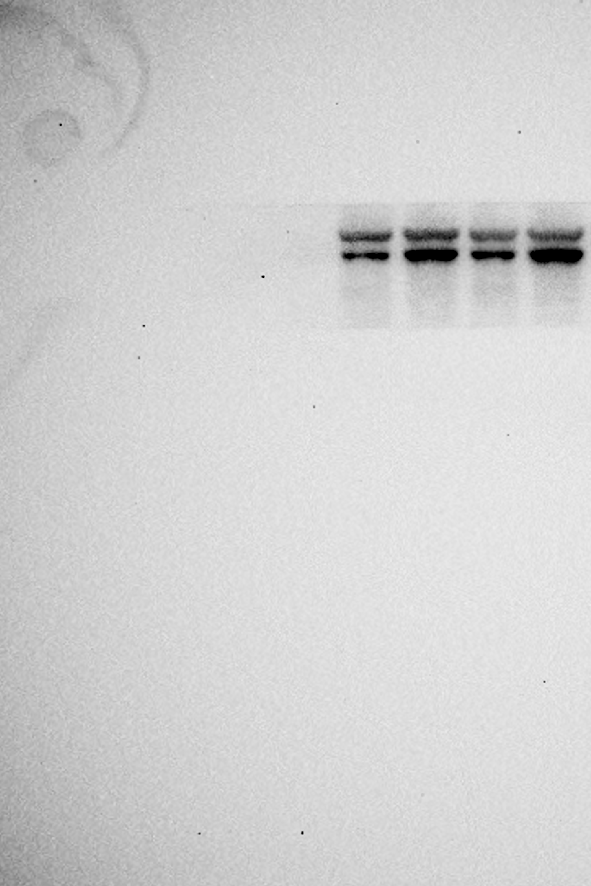

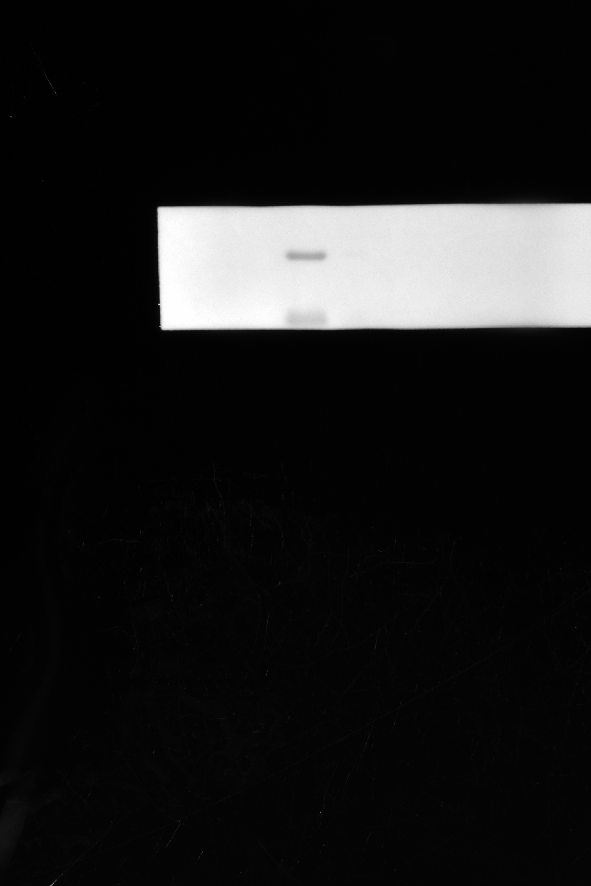

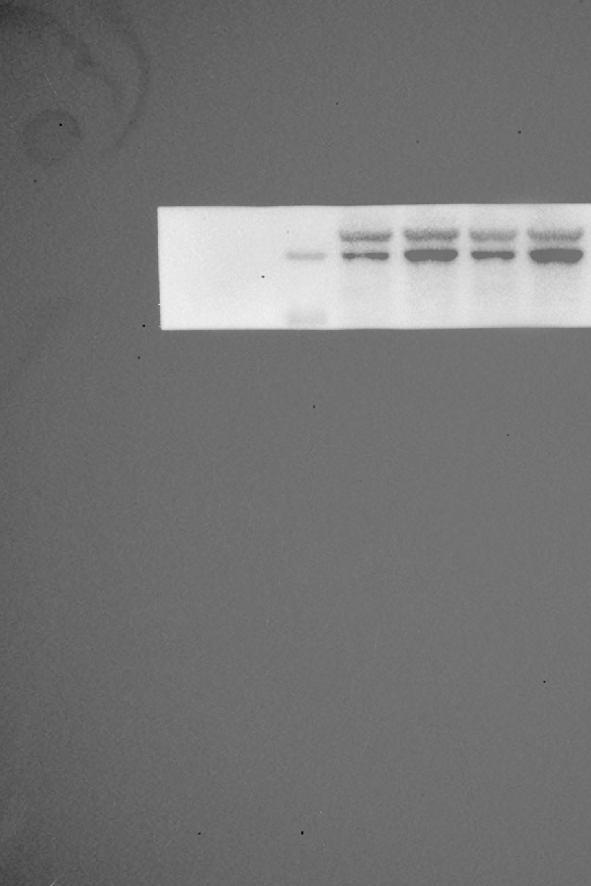

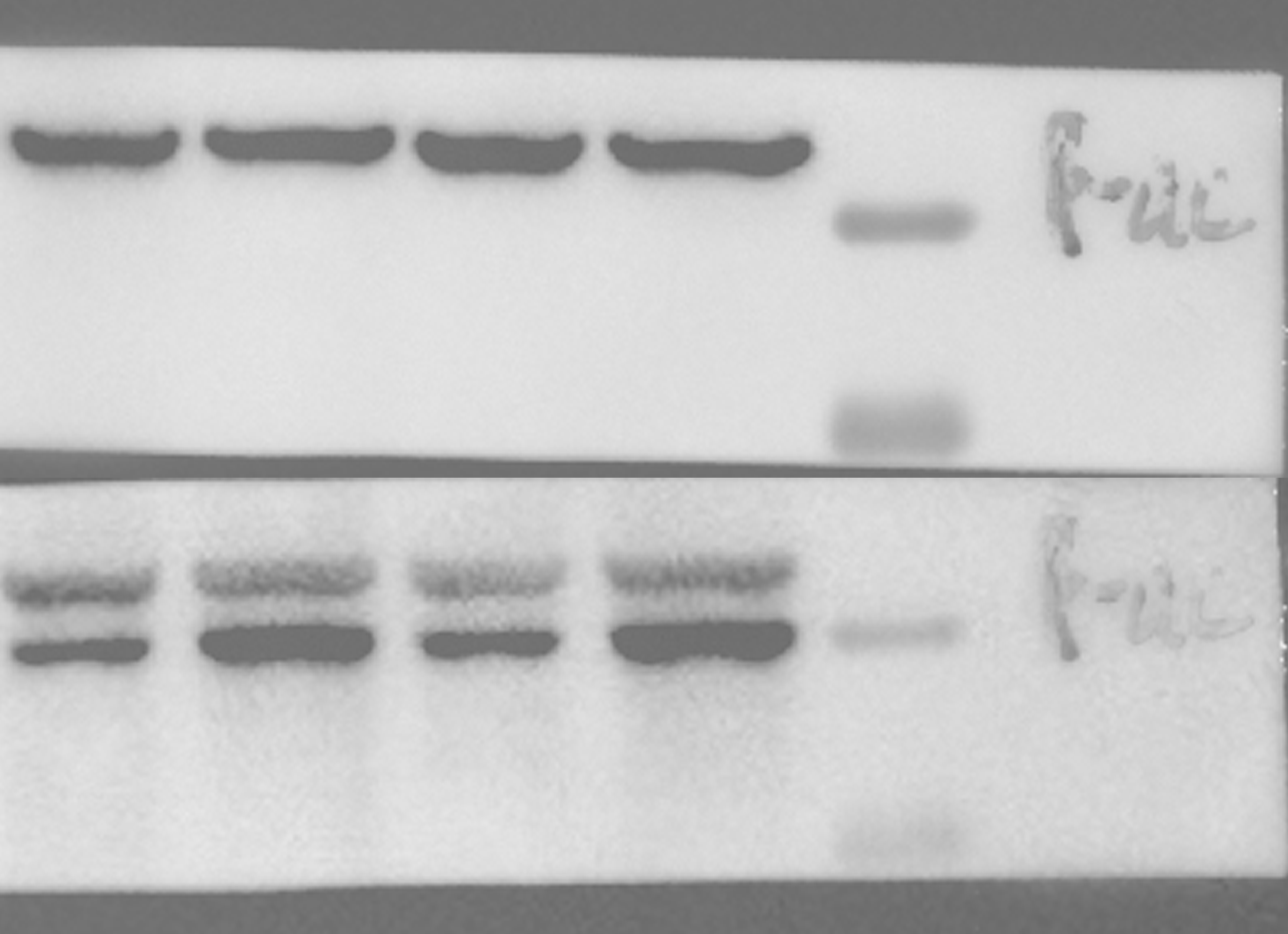


3^rd^ beta-actin_Chemiluminescence → beta-actin_brightfield → beta-actin_merge


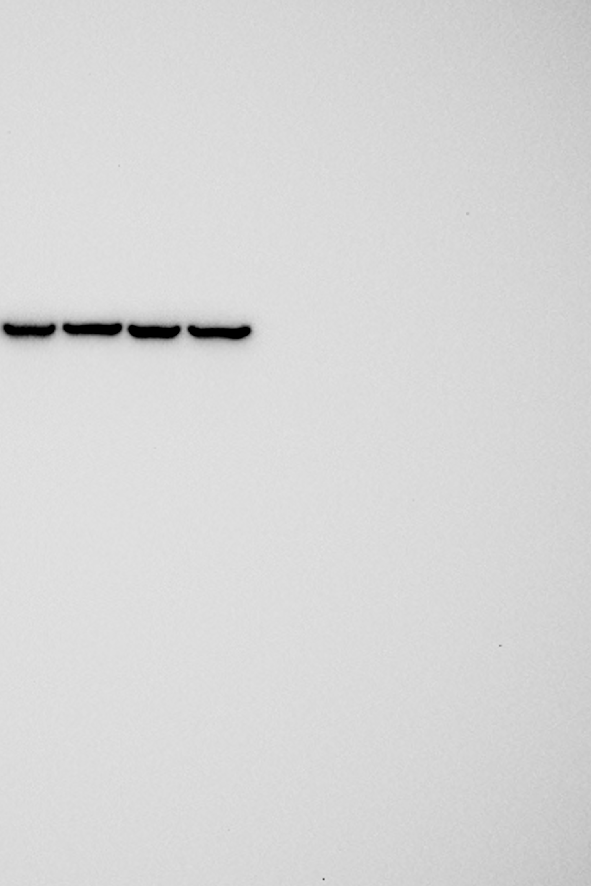

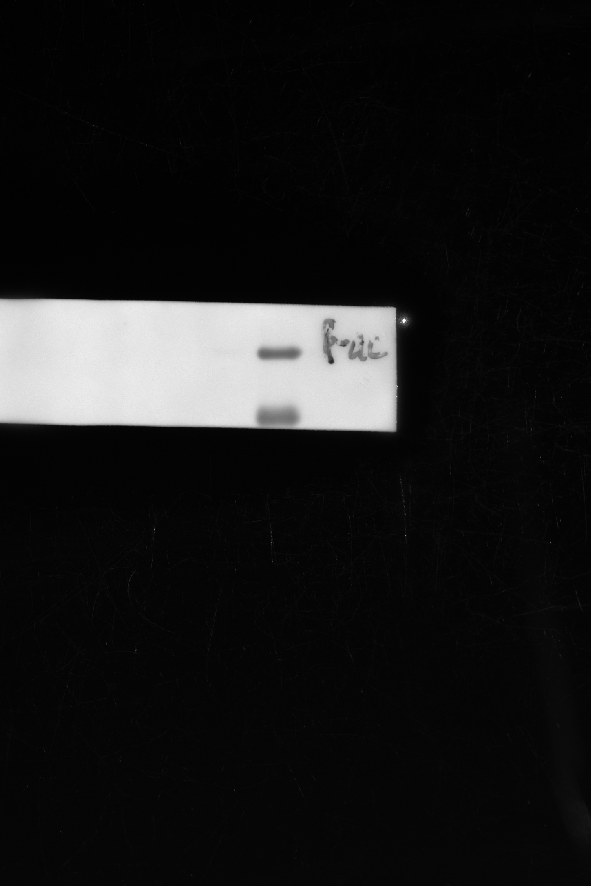

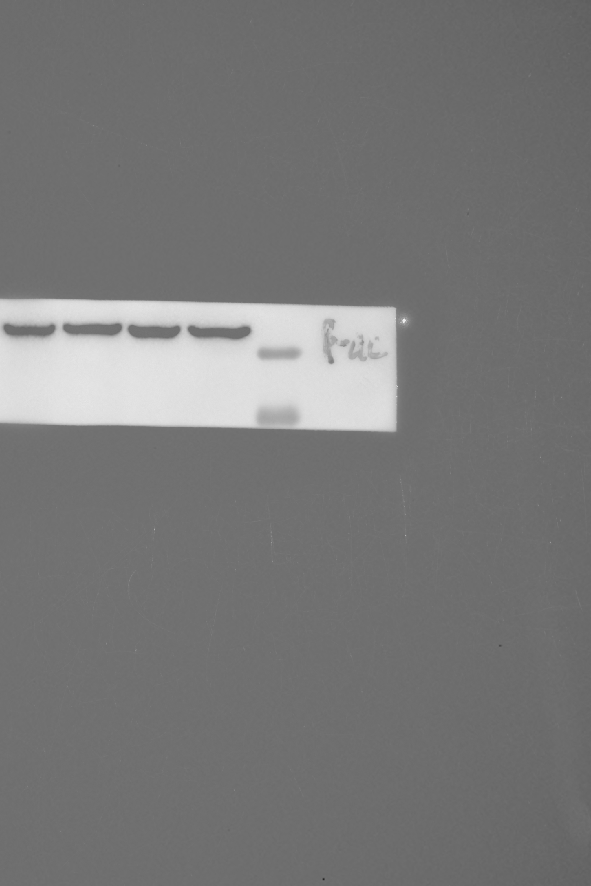


3^rd^ ADH1A_Chemiluminescence → ADH1A_brightfield → ADH1A_merge → ADH1A_beta-actin_complication


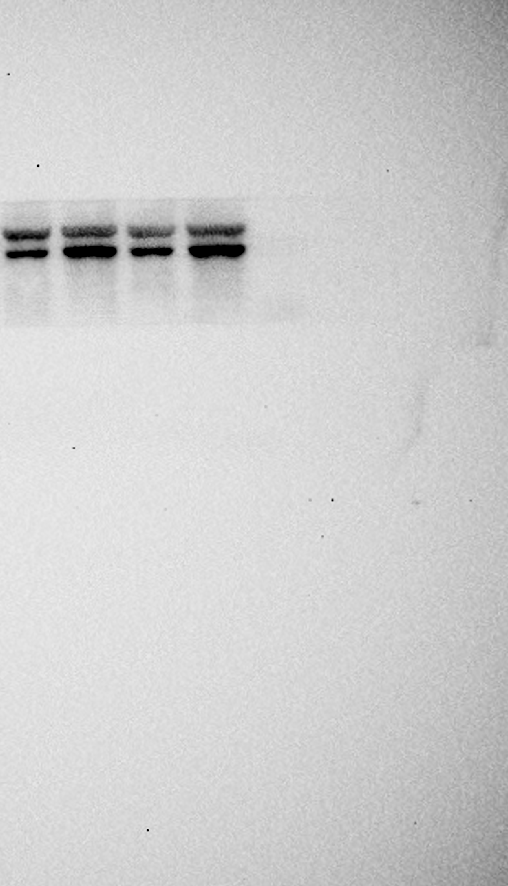

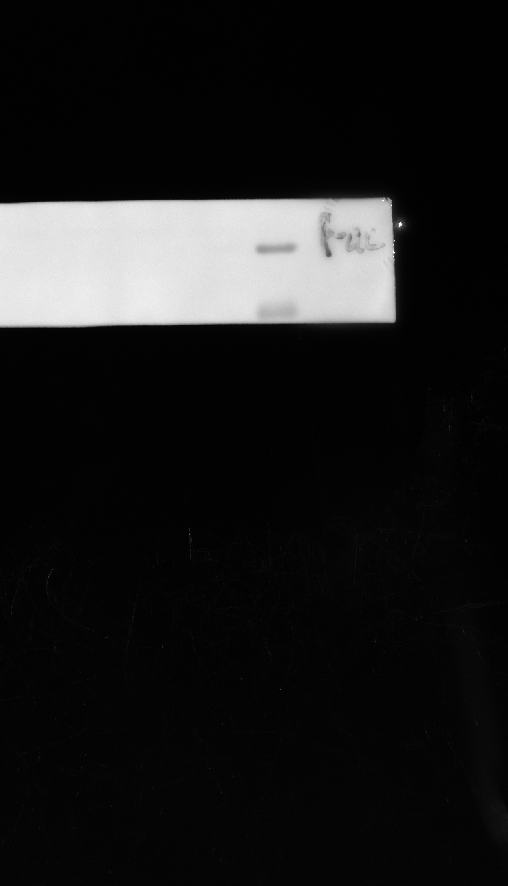

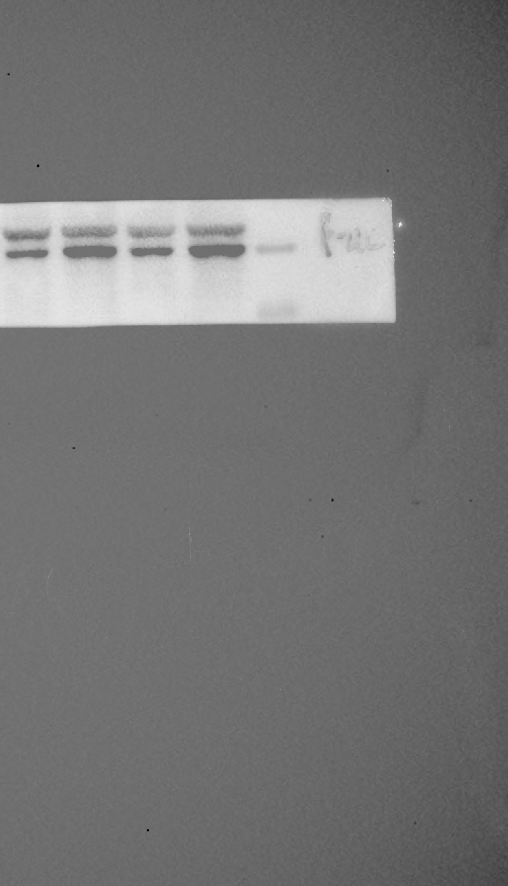

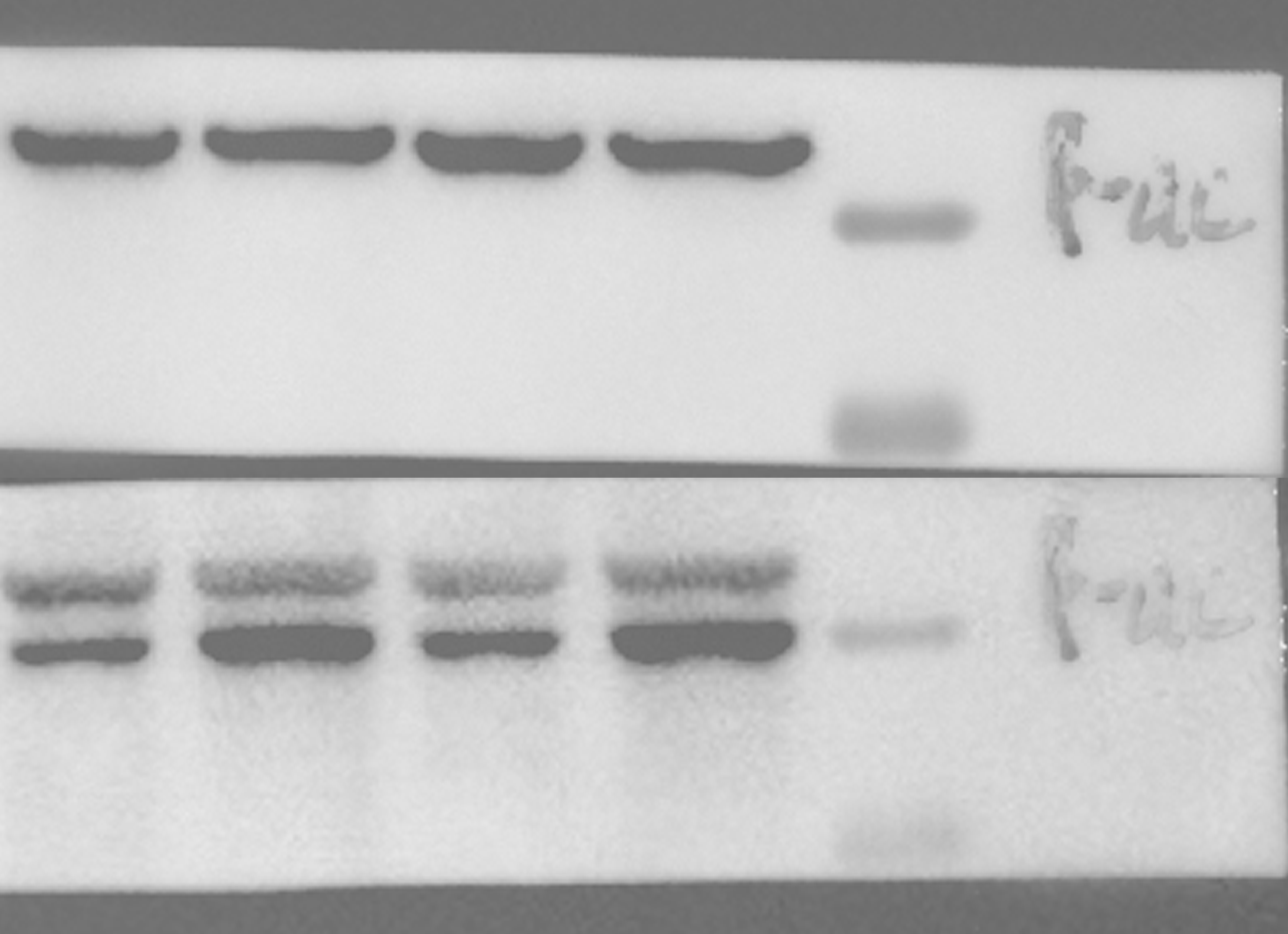


**CDK1**

1^st^ beta-actin_Chemiluminescence → beta-actin_brightfield → beta-actin_merge


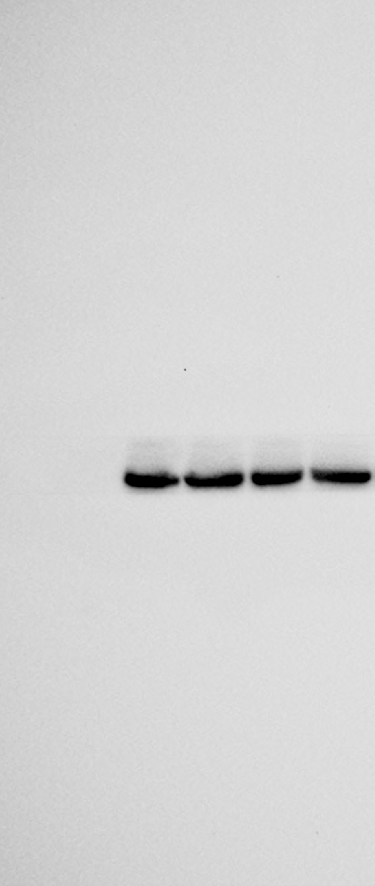

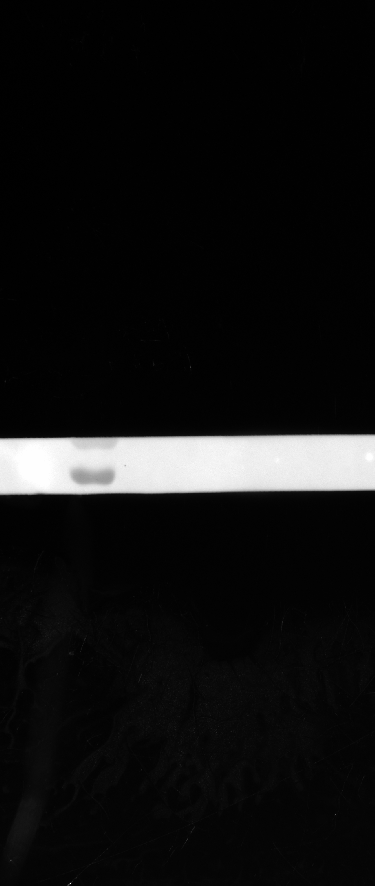

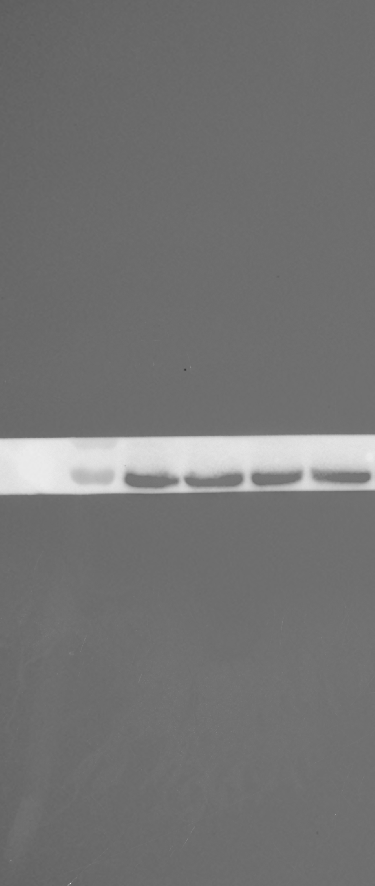


1^st^ CDK1_Chemiluminescence → CDK1_brightfield → CDK1_merge → CDK1_beta-actin_complication


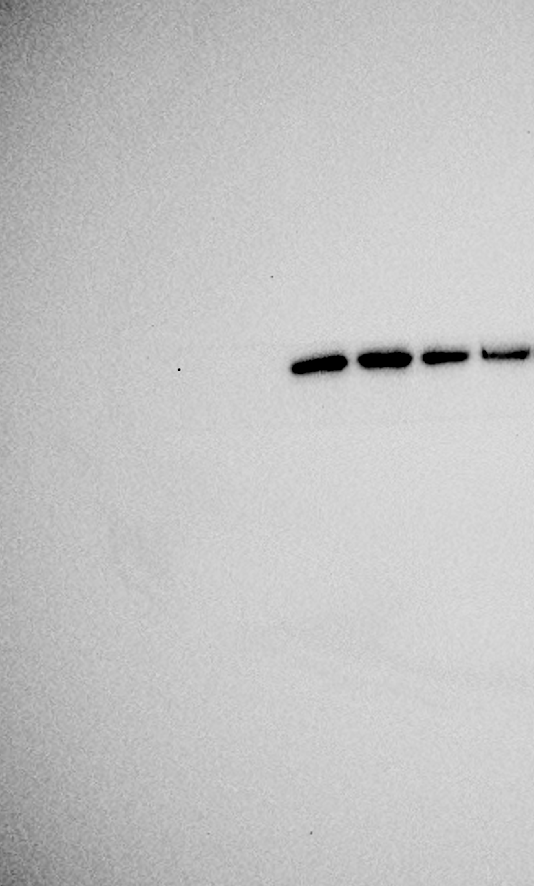

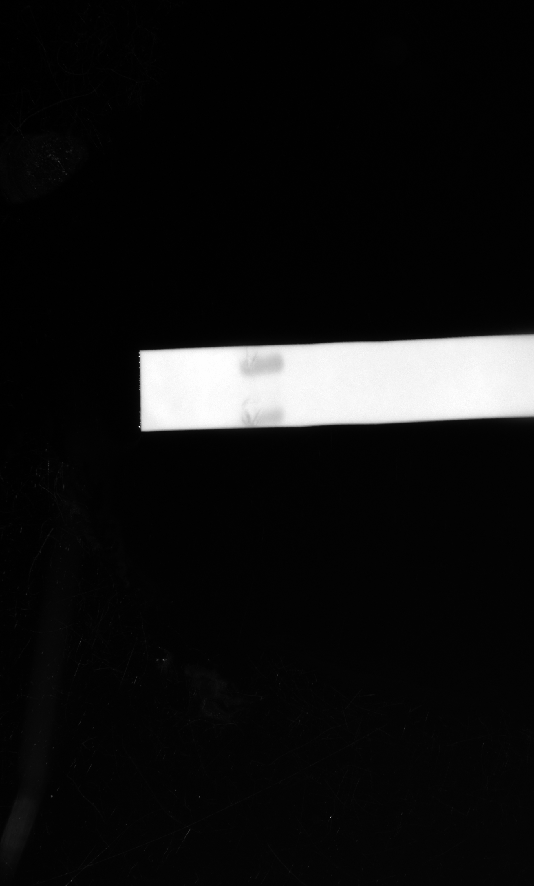

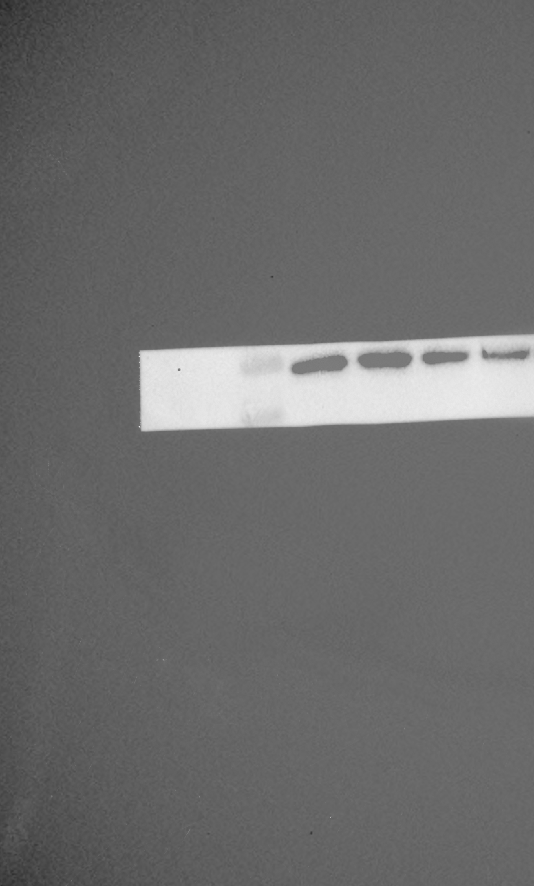

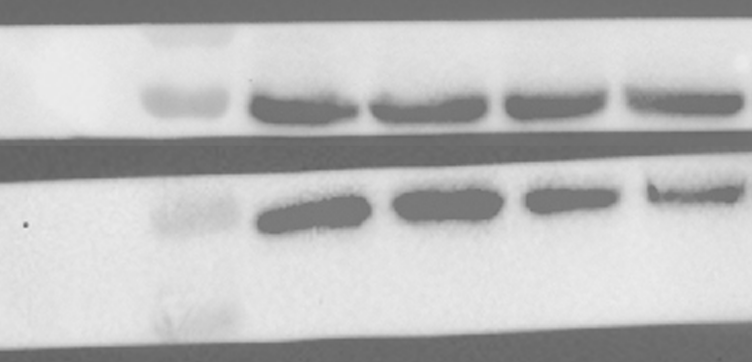


2^nd^ beta-actin_Chemiluminescence → beta-actin_brightfield → beta-actin_merge


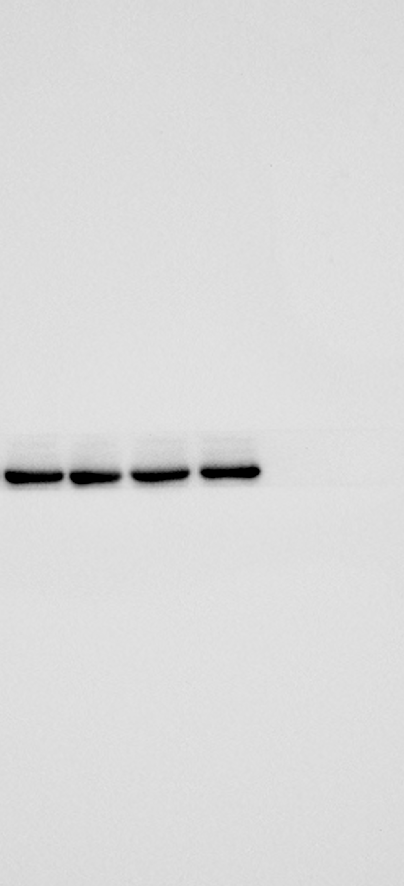

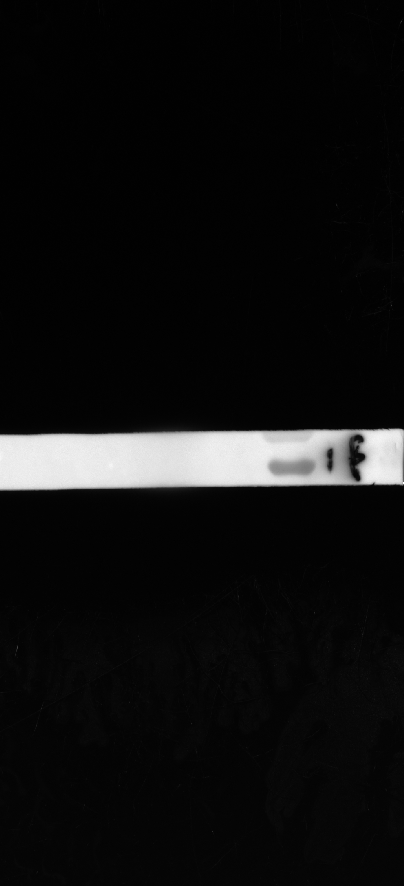

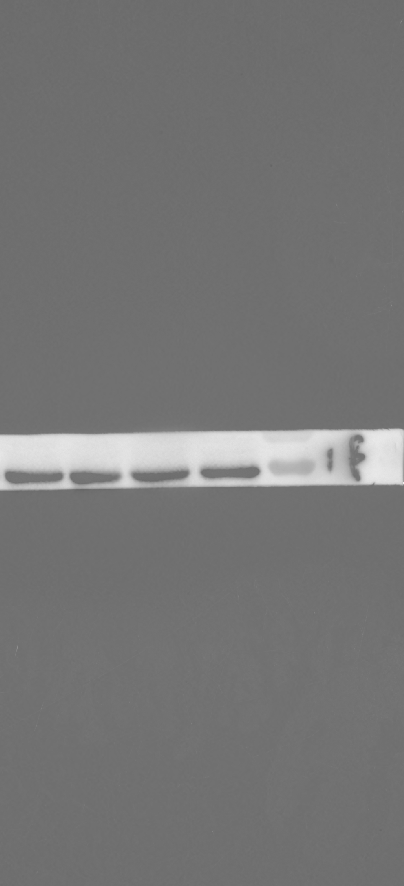


2^nd^ CDK1_Chemiluminescence → CDK1_brightfield → CDK1_merge → CDK1_beta-actin_complication


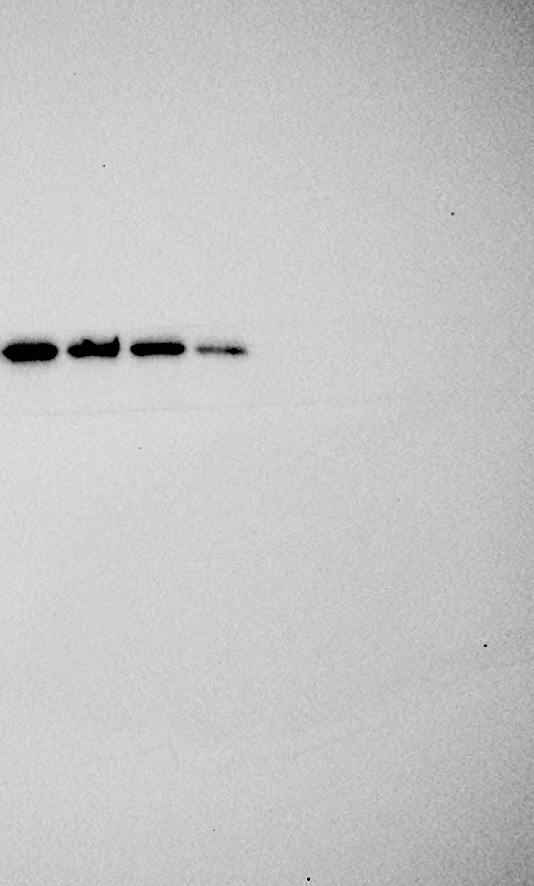

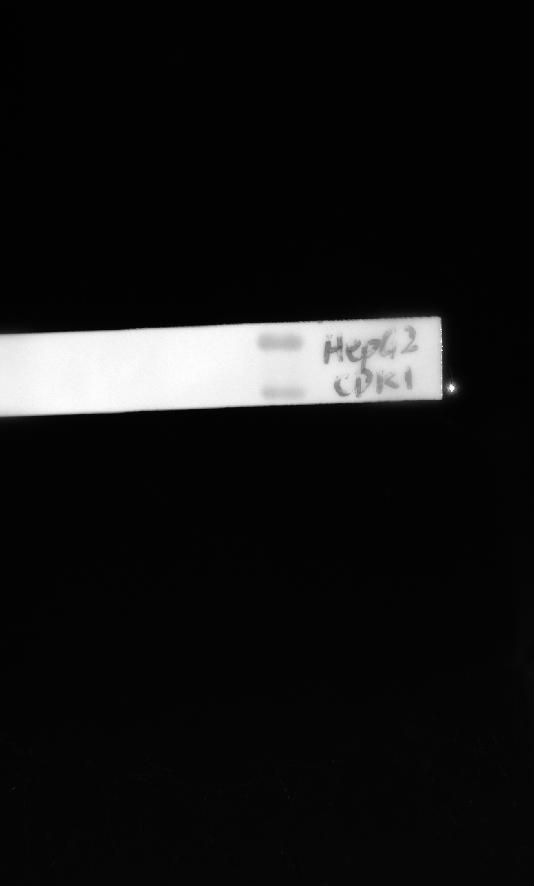

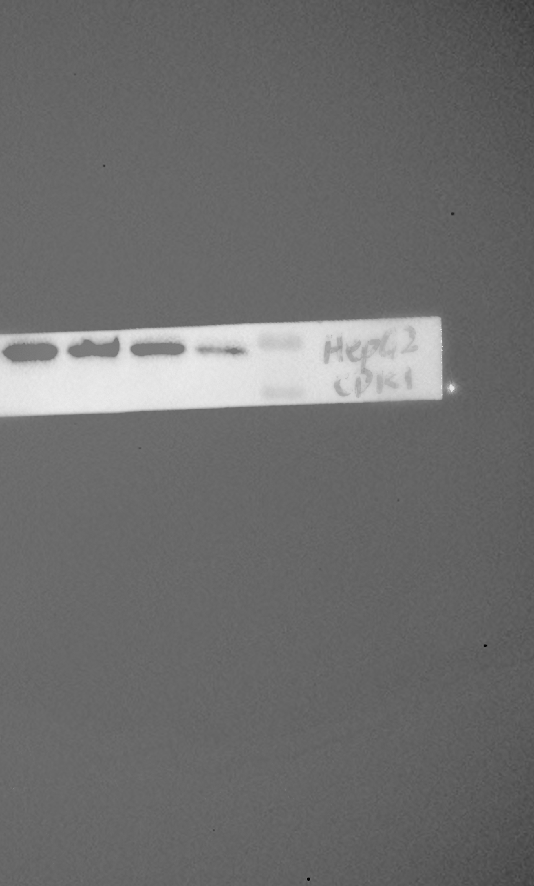

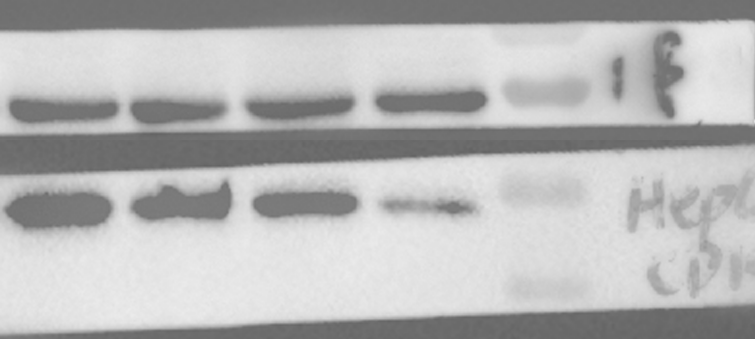


3^rd^ beta-actin_Chemiluminescence → beta-actin_brightfield → beta-actin_merge


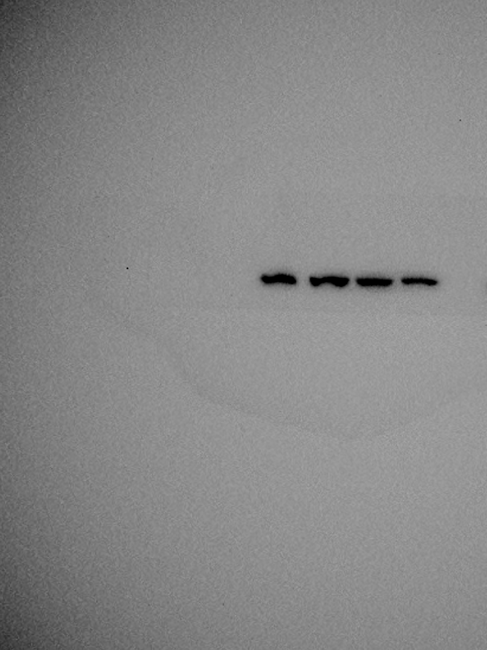

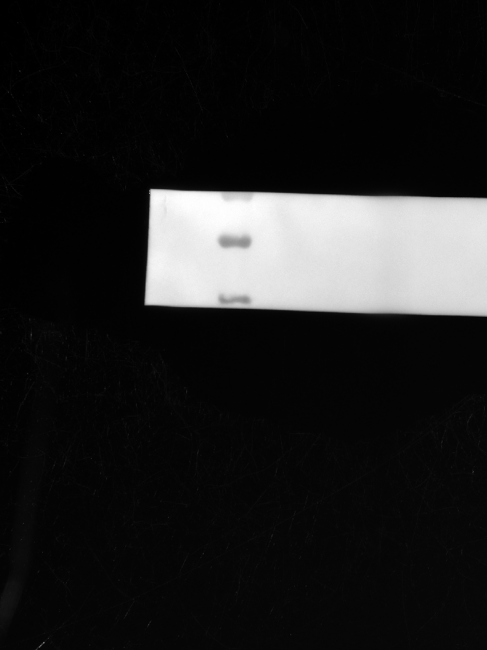

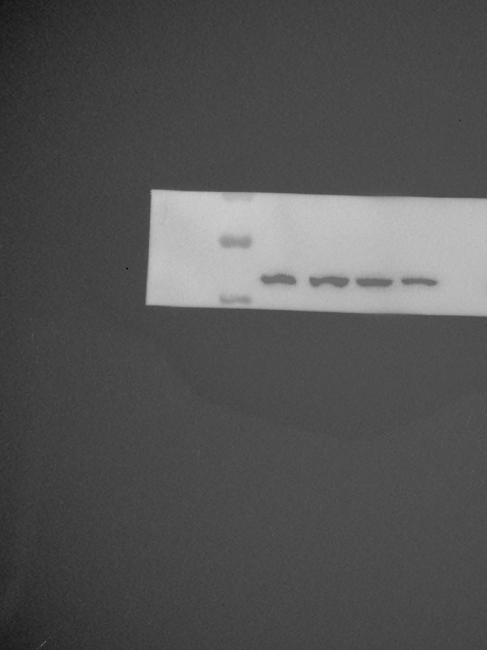


3^rd^ CDK1_Chemiluminescence → CDK1_brightfield → CDK1_merge → CDK1_beta-actin_complication


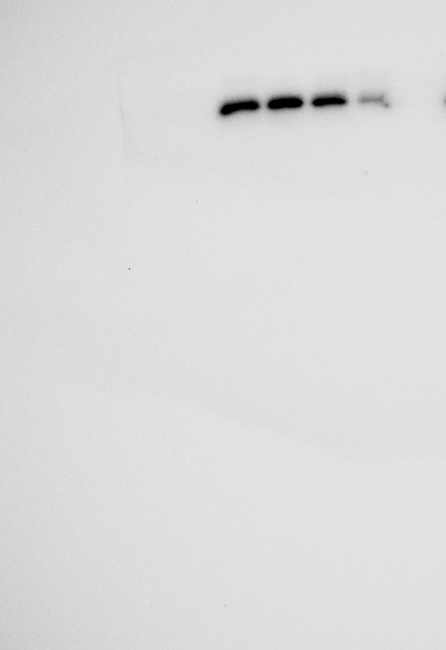

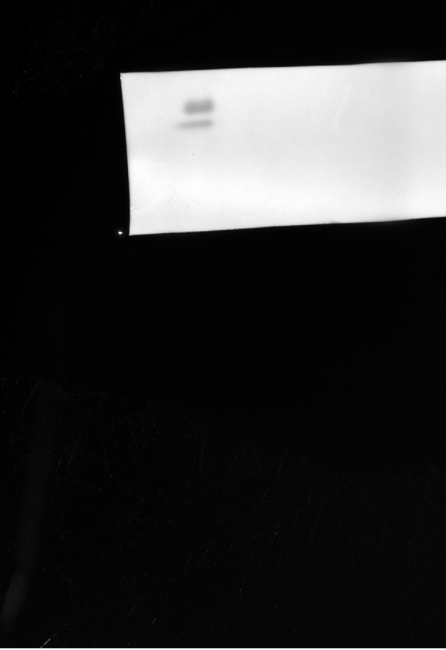

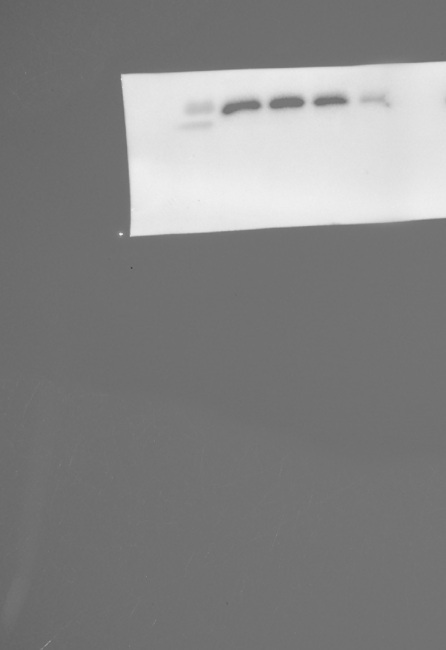

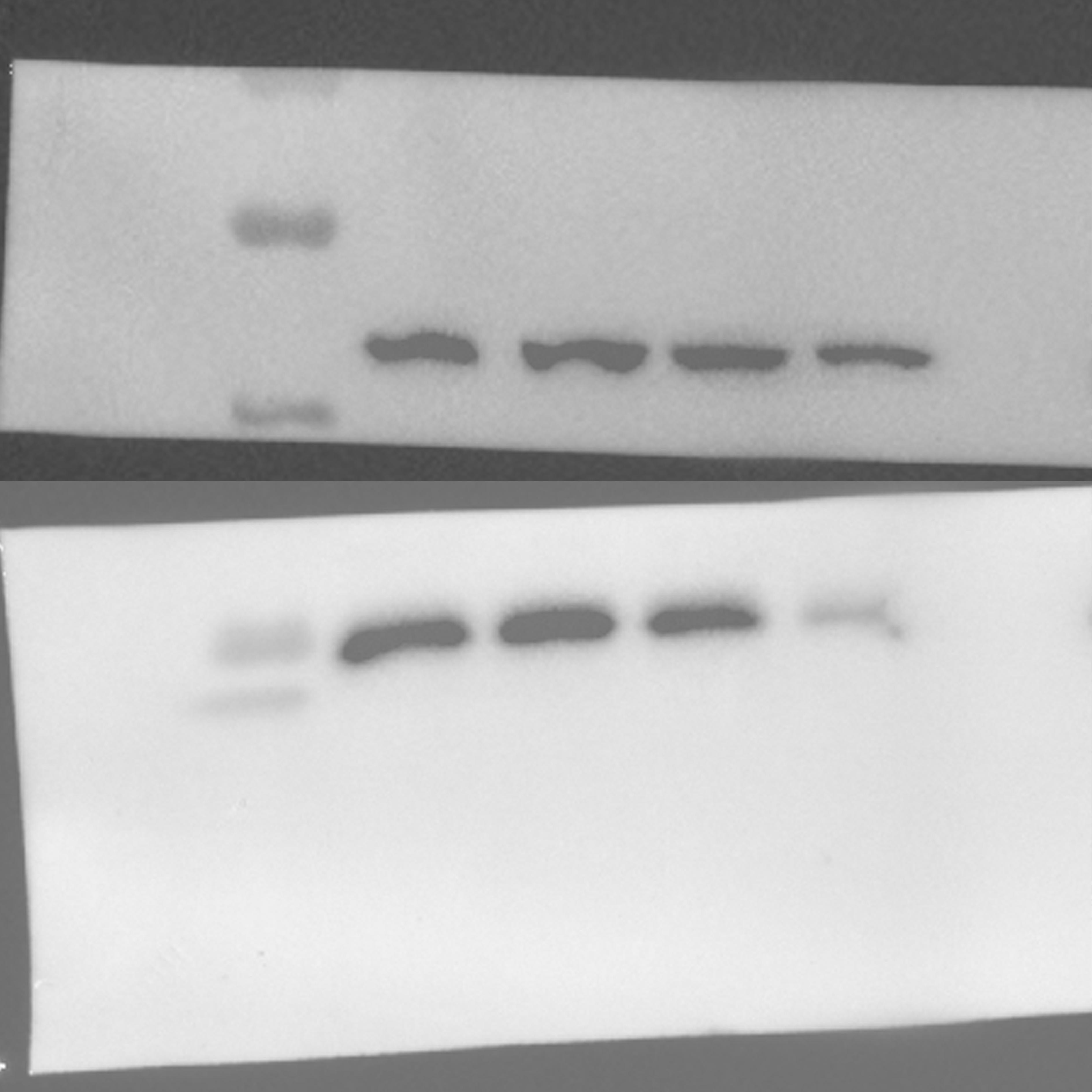


**EPHX2**

1^st^ beta-actin_Chemiluminescence → beta-actin_brightfield → beta-actin_merge


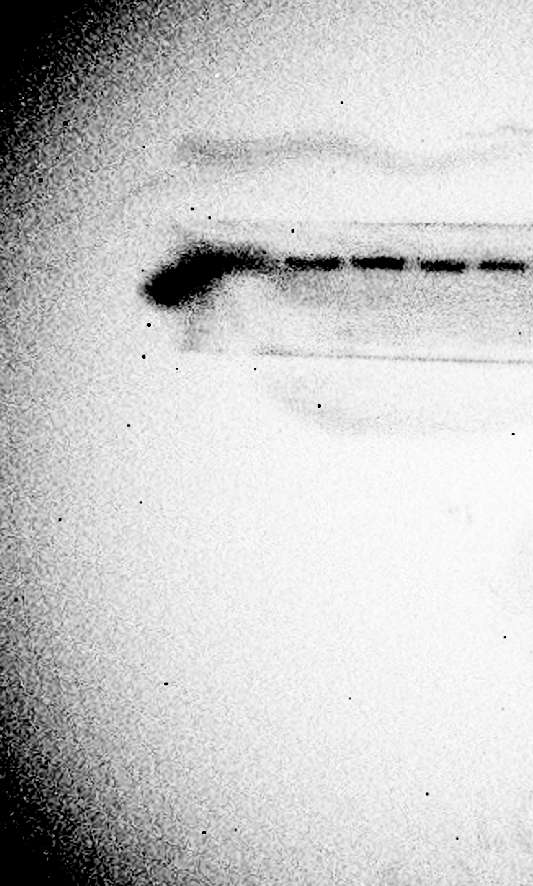

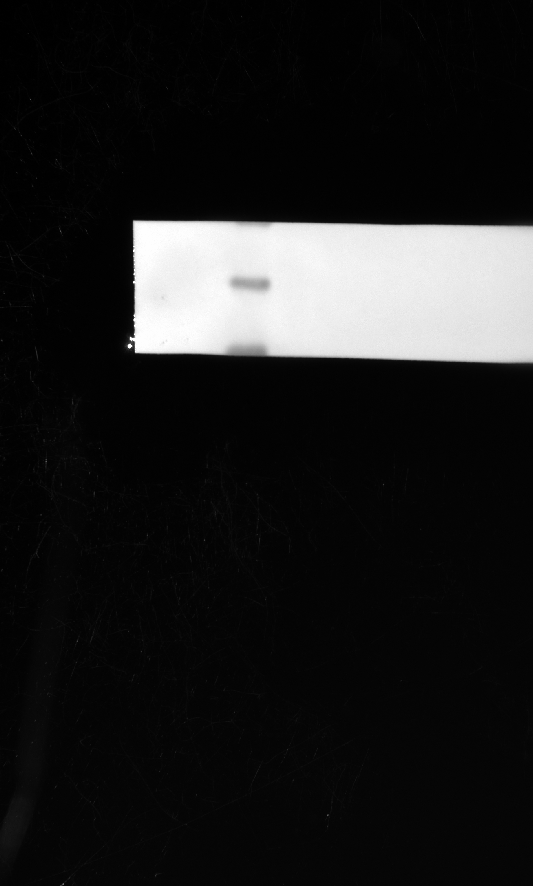

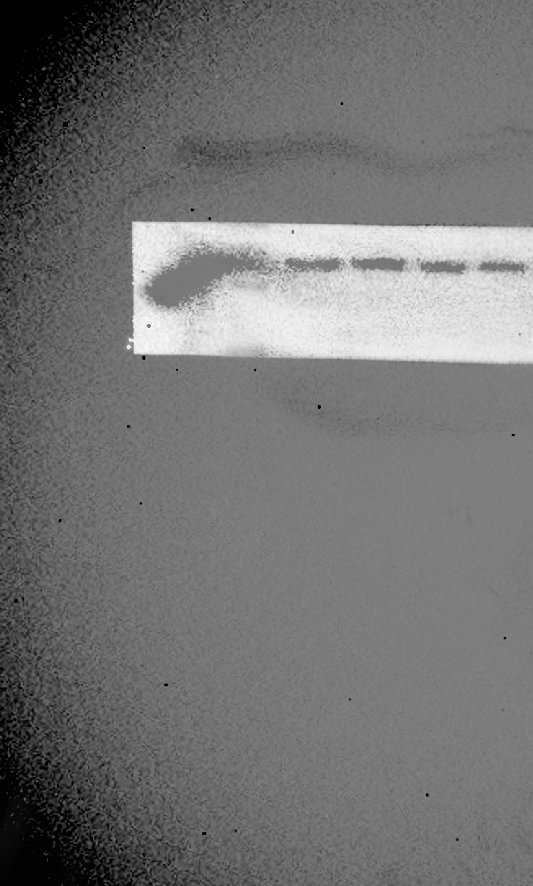


1^st^ EPHX2_Chemiluminescence → EPHX2_brightfield → EPHX2_merge → EPHX2_beta-actin_complication


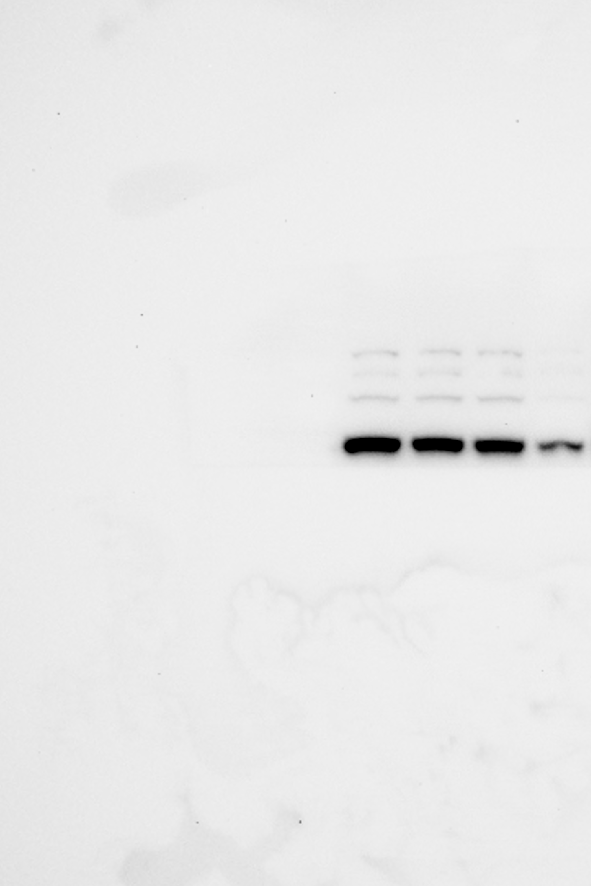

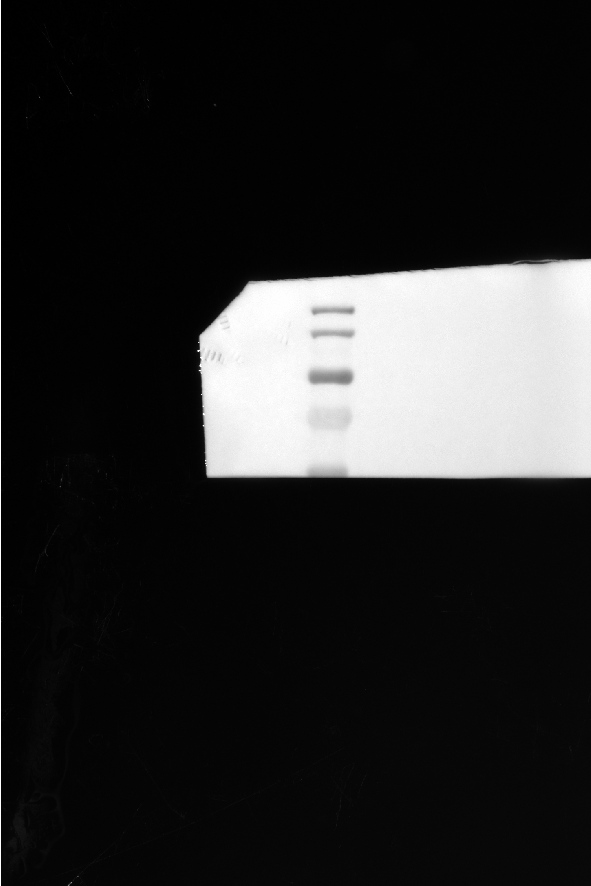

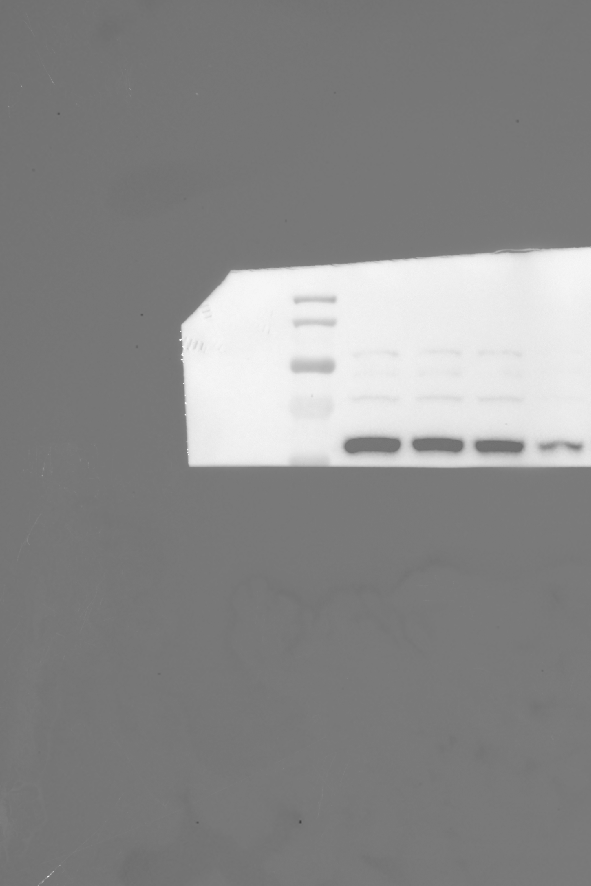

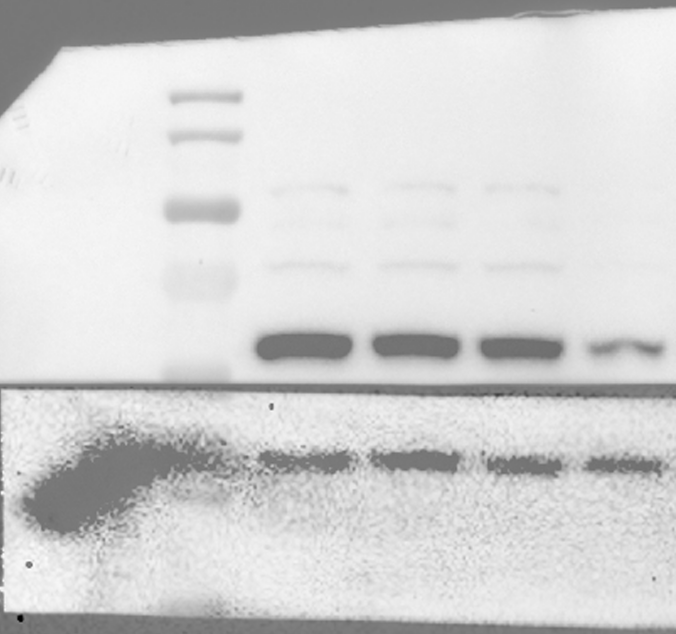


2^nd^ beta-actin_Chemiluminescence → beta-actin_brightfield → beta-actin_merge


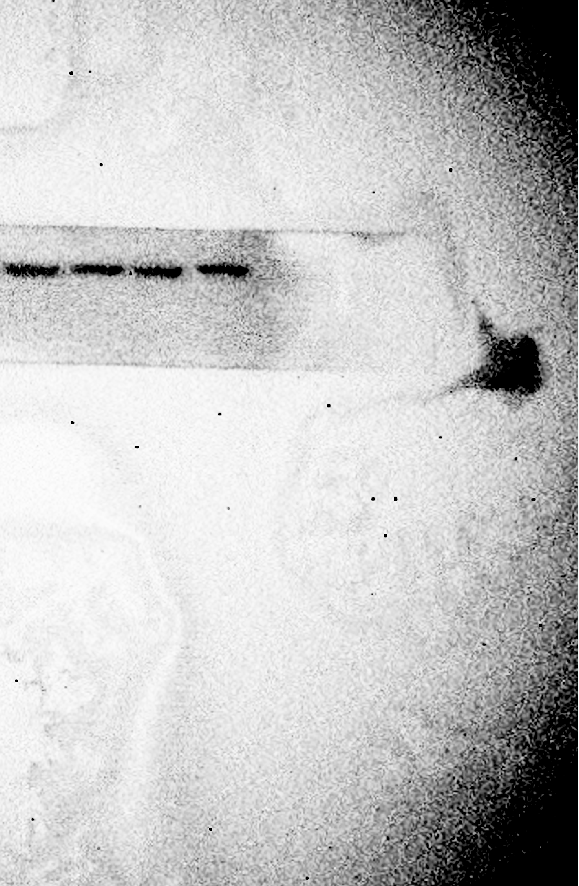

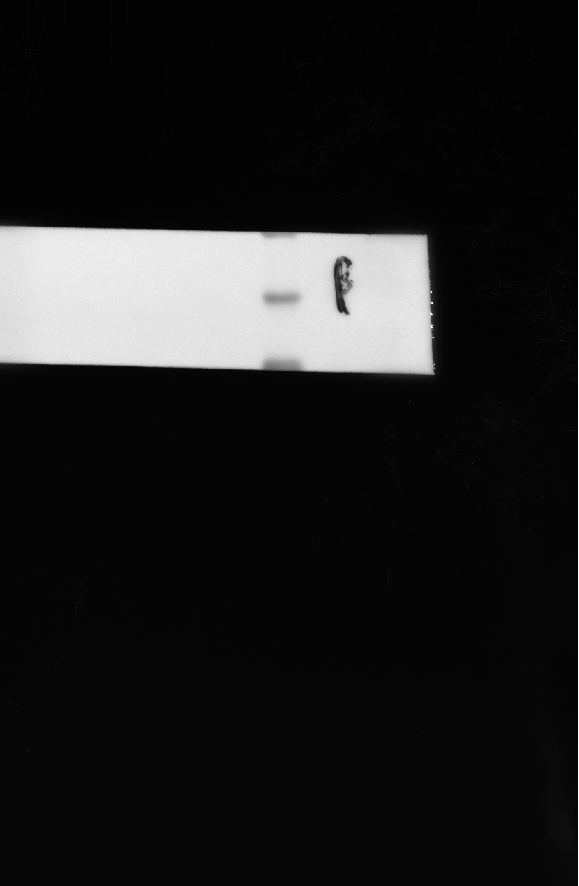

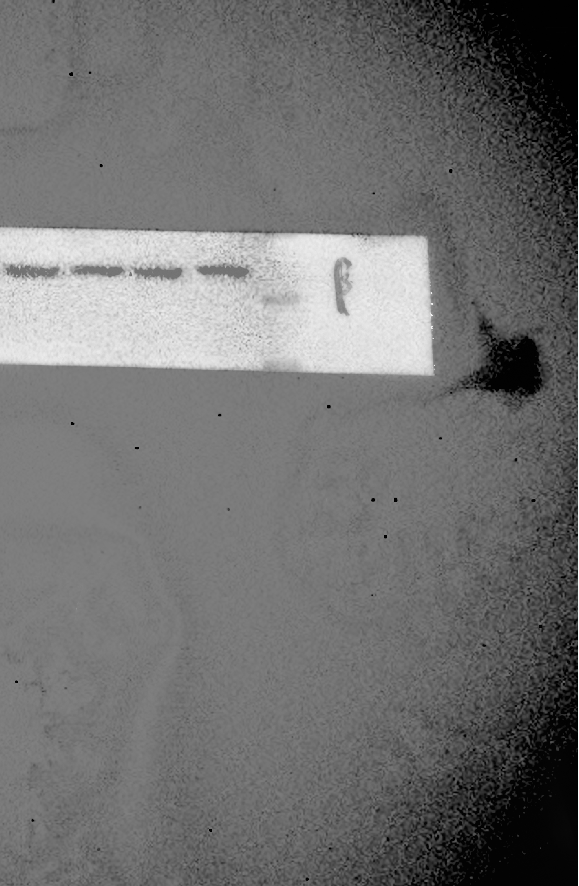


2^nd^ EPHX2_Chemiluminescence → EPHX2_brightfield → EPHX2_merge → EPHX2_beta-actin_complication


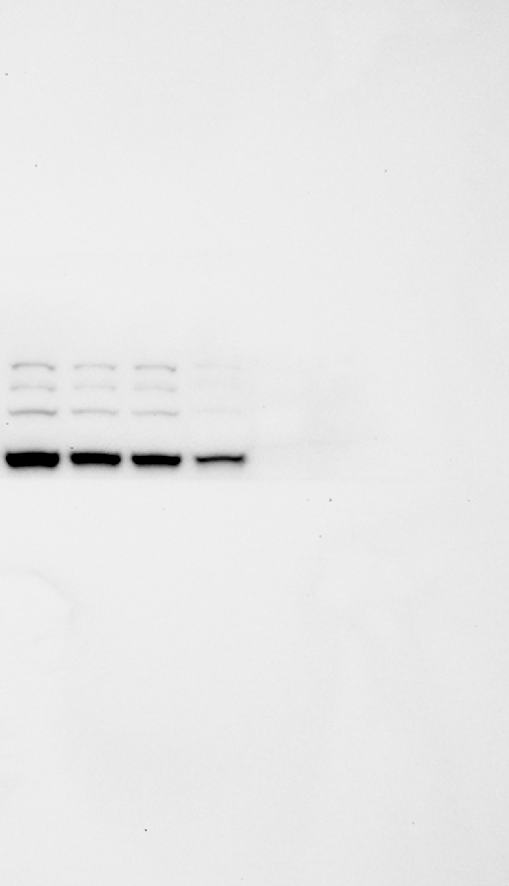

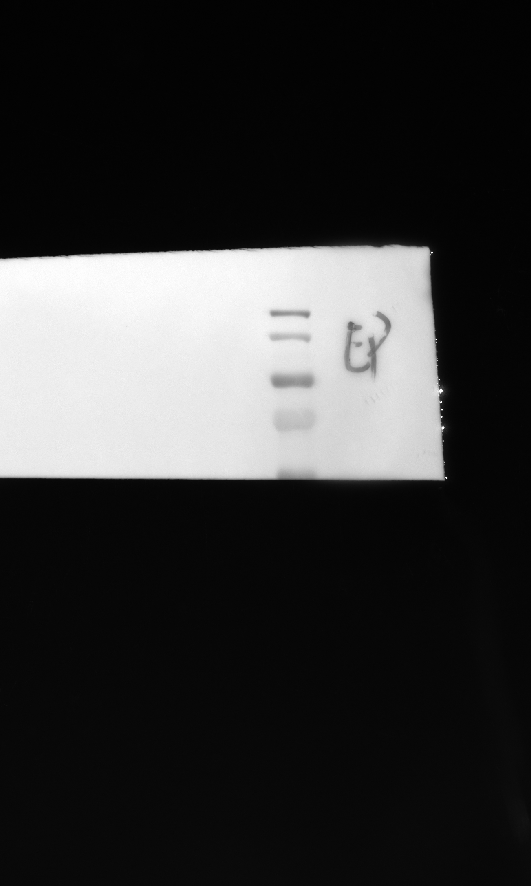

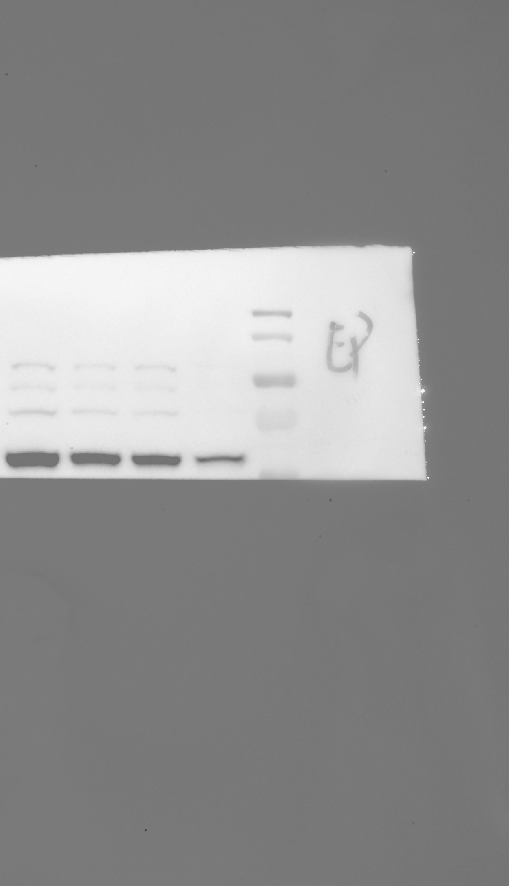

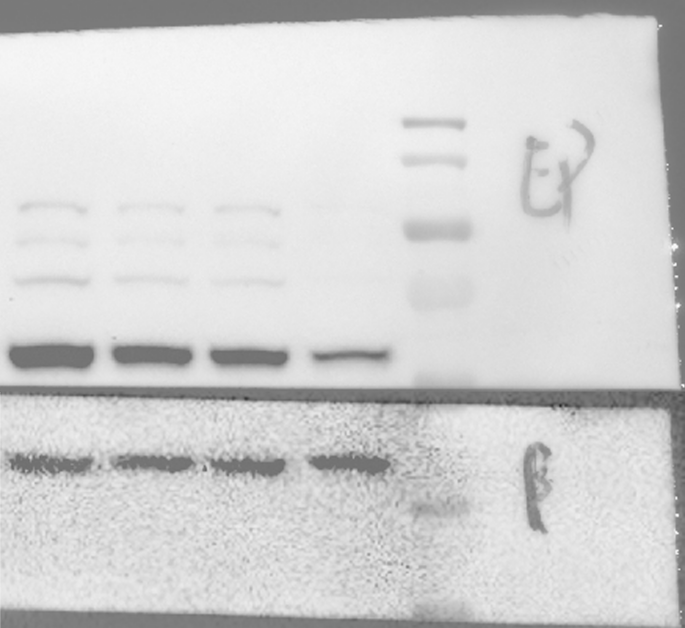


3^rd^ beta-actin_Chemiluminescence → beta-actin_brightfield → beta-actin_merge


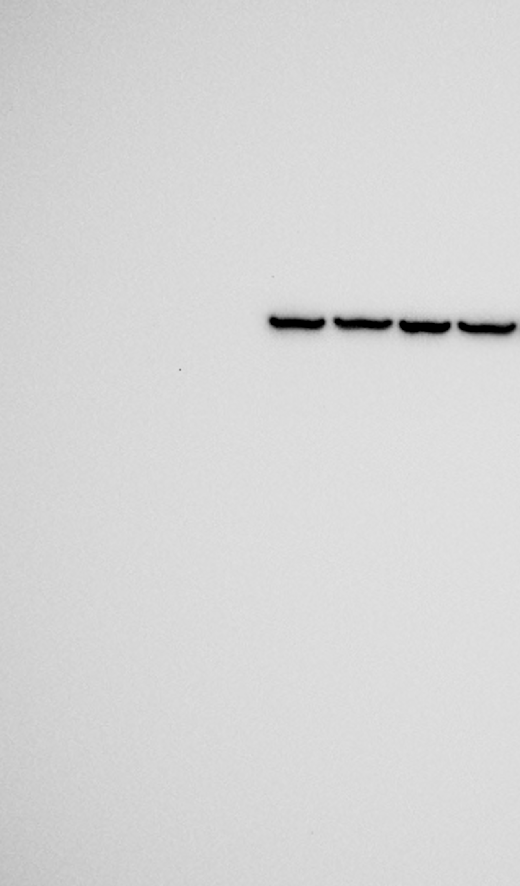

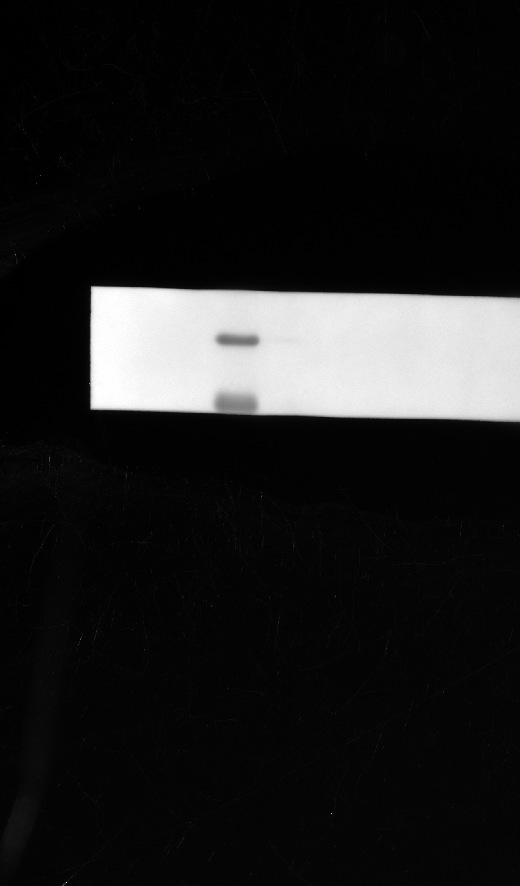

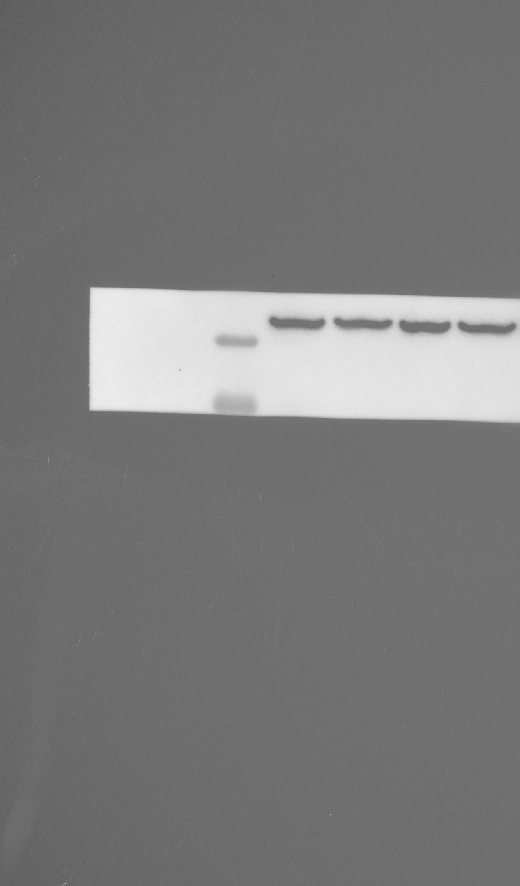


3^rd^ EPHX2_Chemiluminescence → EPHX2_brightfield → EPHX2_merge → EPHX2_beta-actin_complication


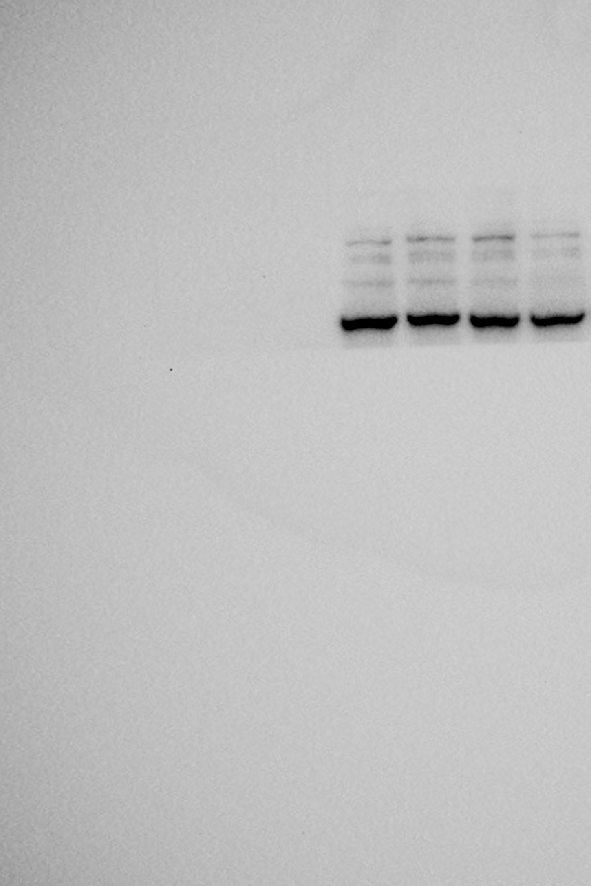

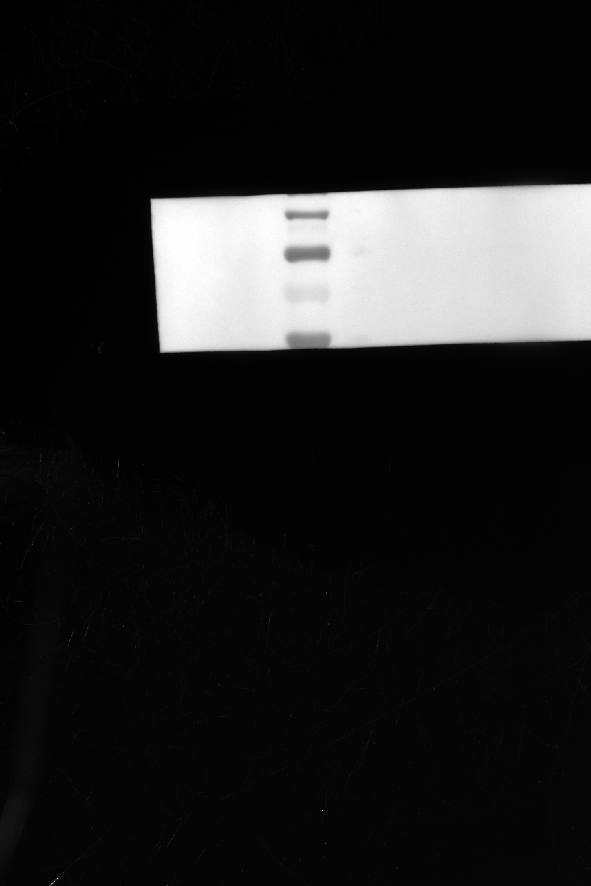

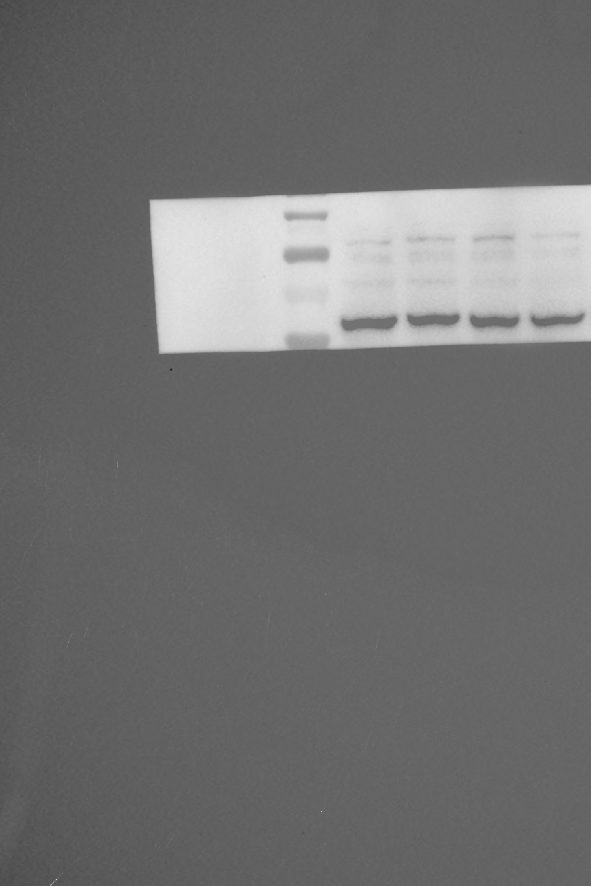

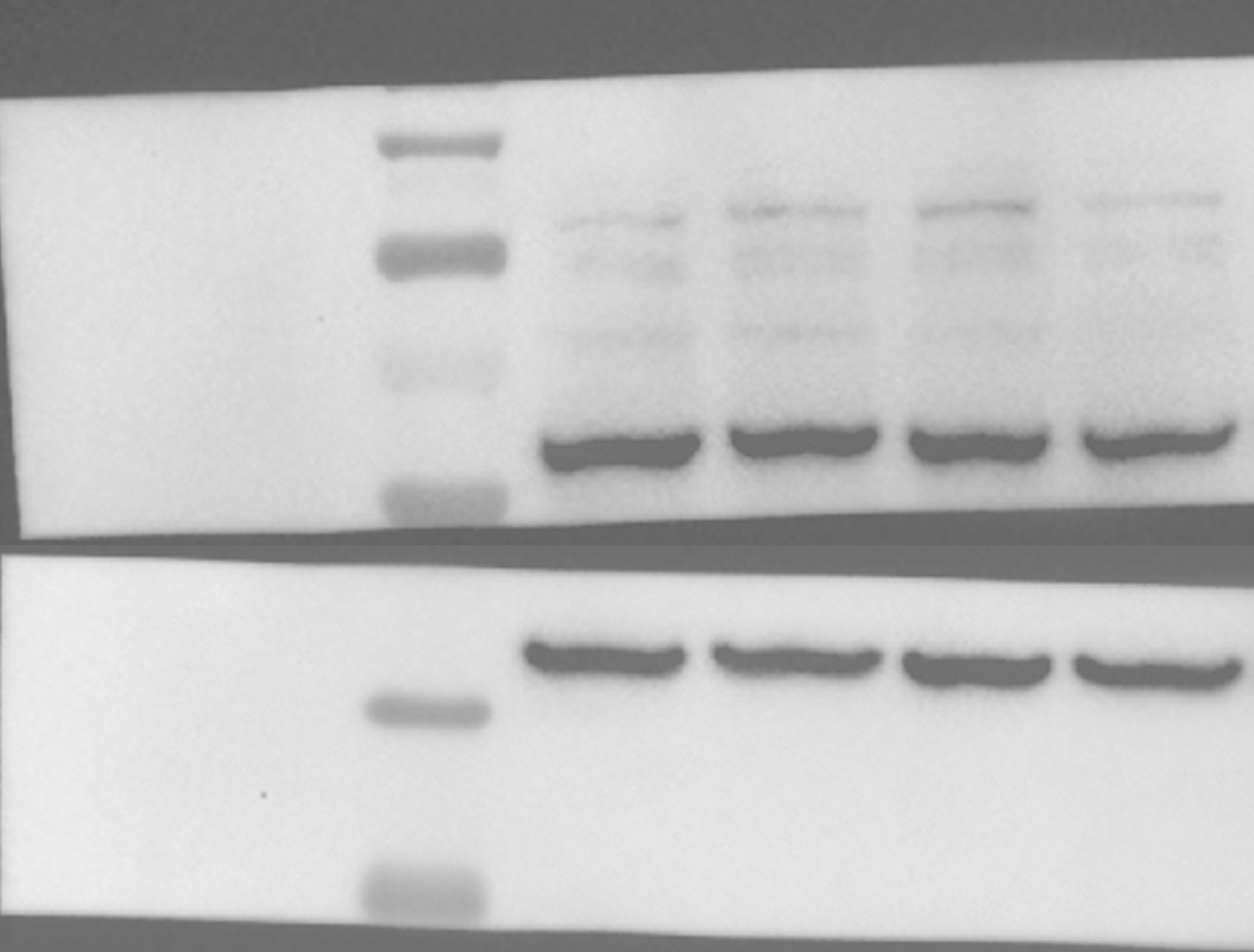


**SRD5A2**

1^st^ beta-actin_Chemiluminescence → beta-actin_brightfield → beta-actin_merge


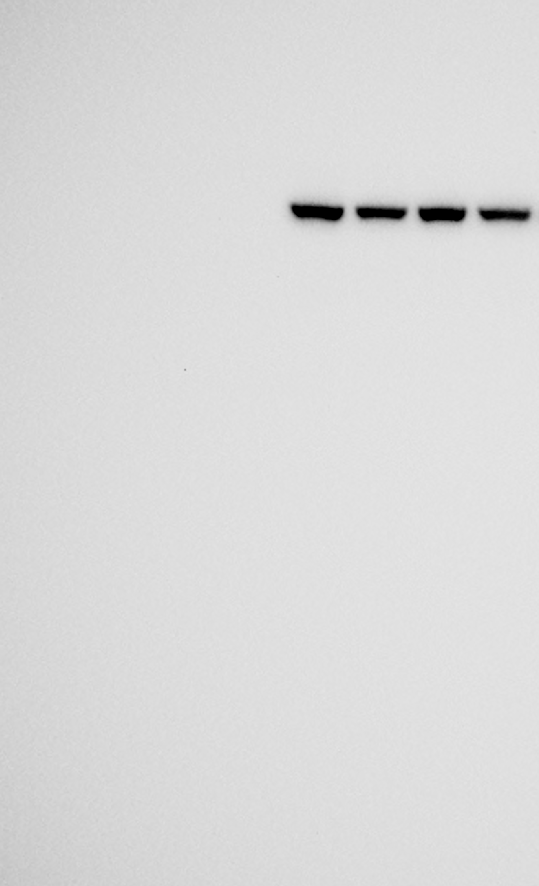

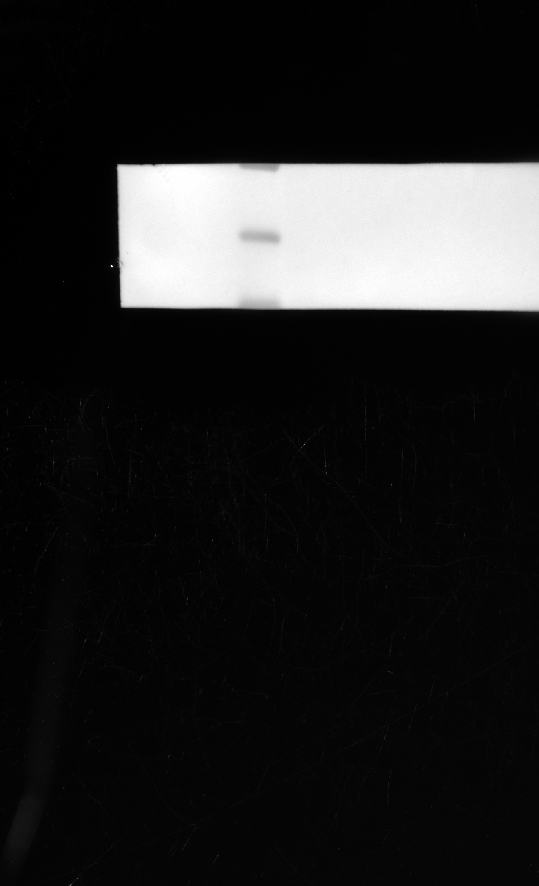

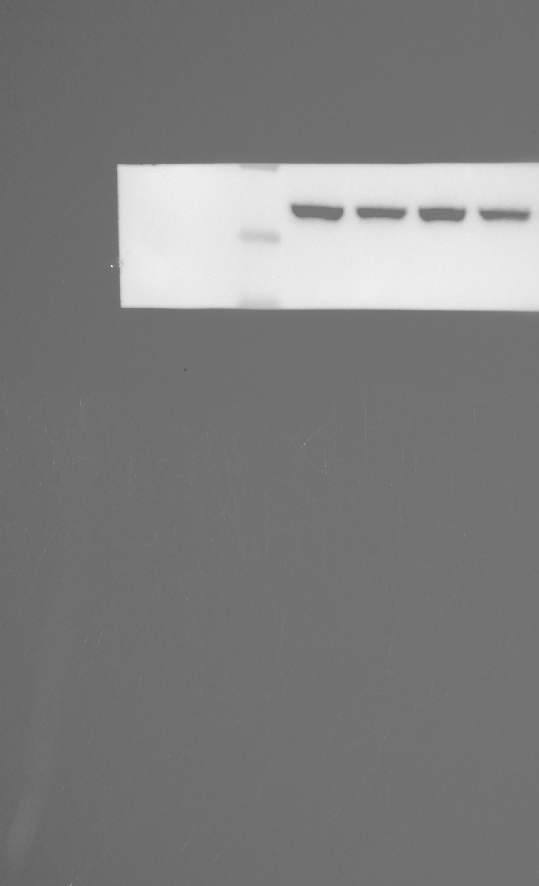


1^st^ SRD5A2_Chemiluminescence → SRD5A2_brightfield → SRD5A2_merge → SRD5A2_beta-actin_complication


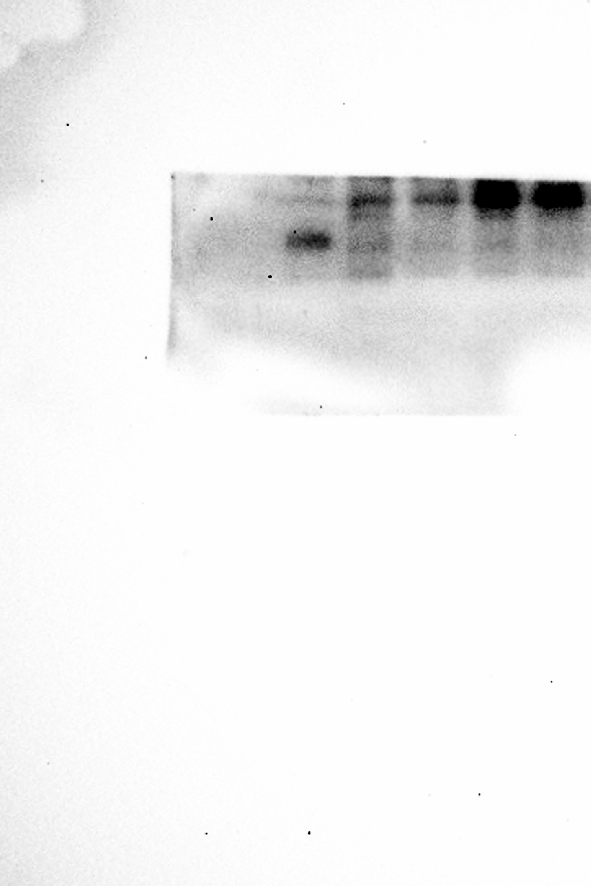

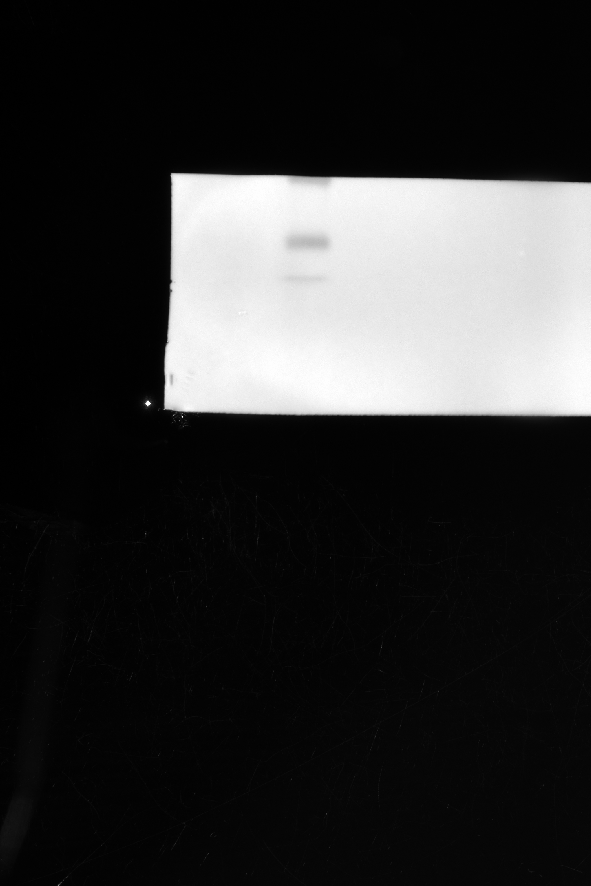

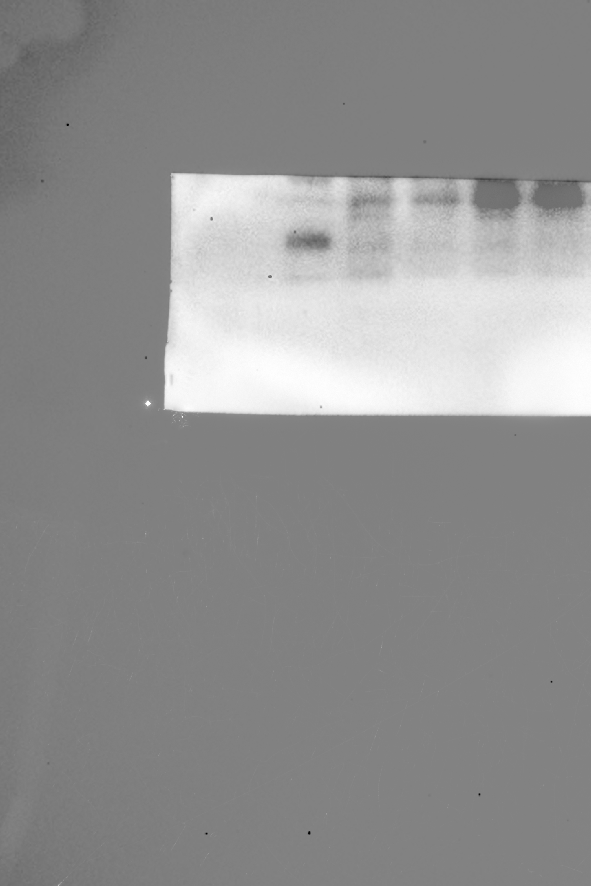

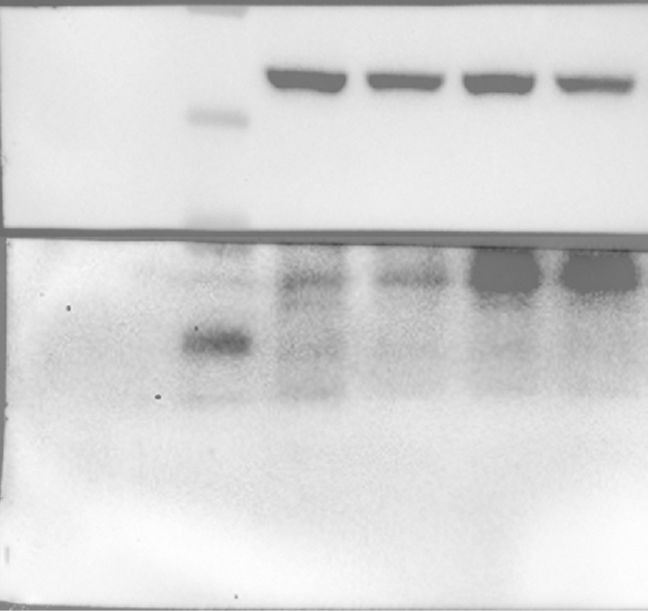


2^nd^ beta-actin_Chemiluminescence → beta-actin_brightfield → beta-actin_merge


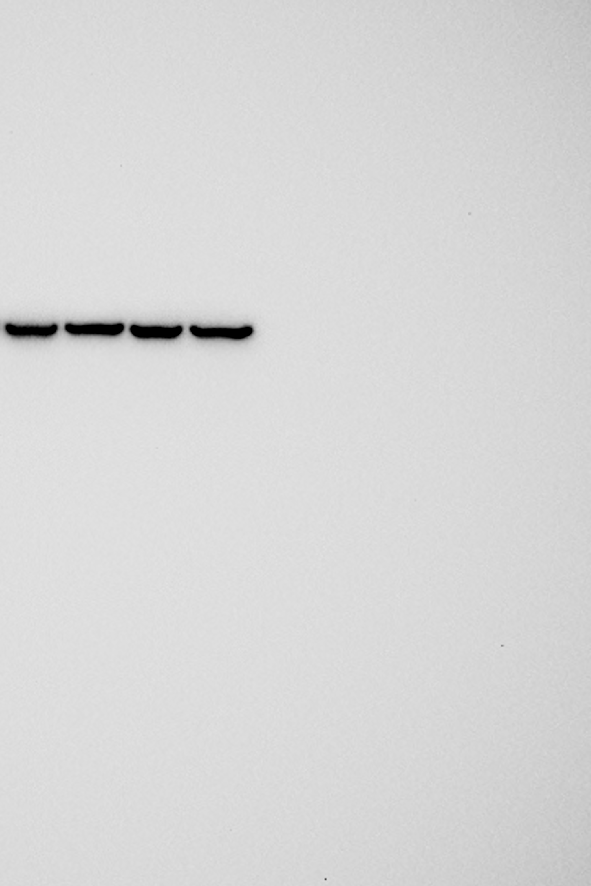

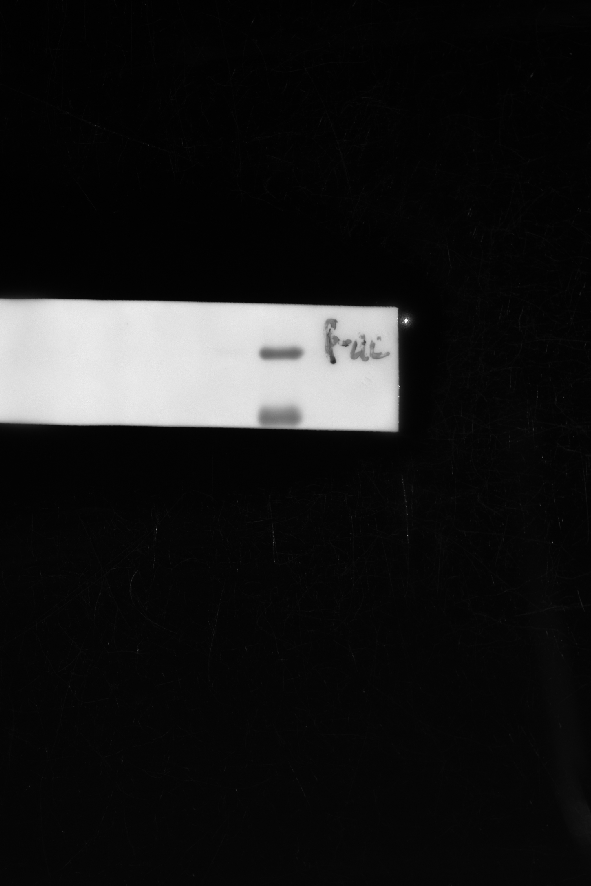

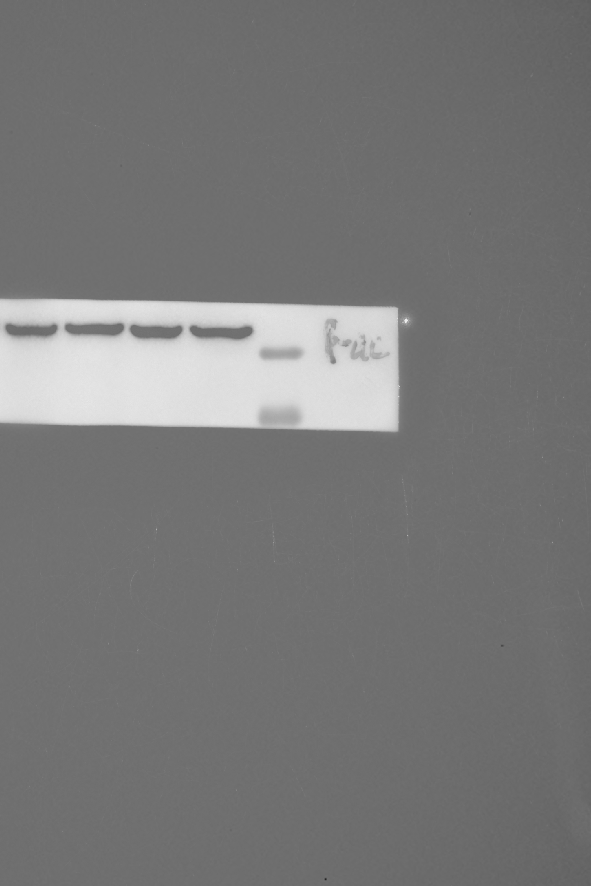


2^nd^ SRD5A2_Chemiluminescence → SRD5A2_brightfield → SRD5A2_merge → SRD5A2_beta-actin_complication


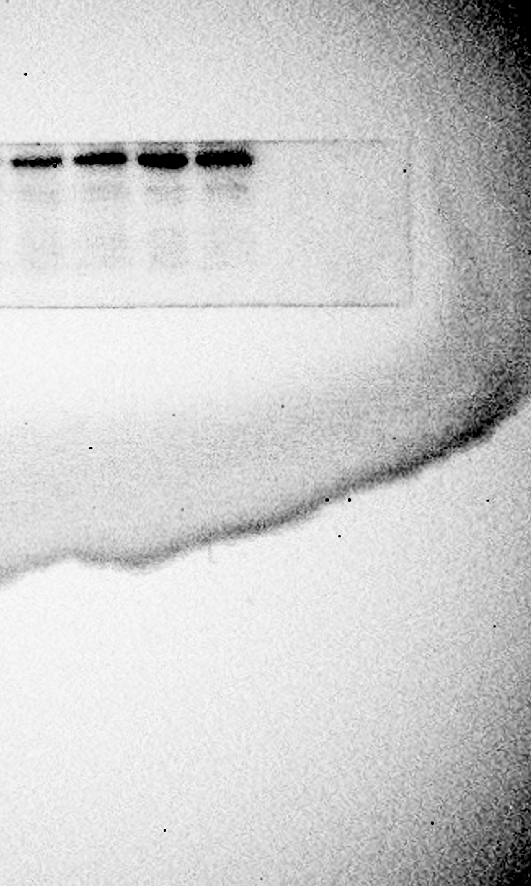

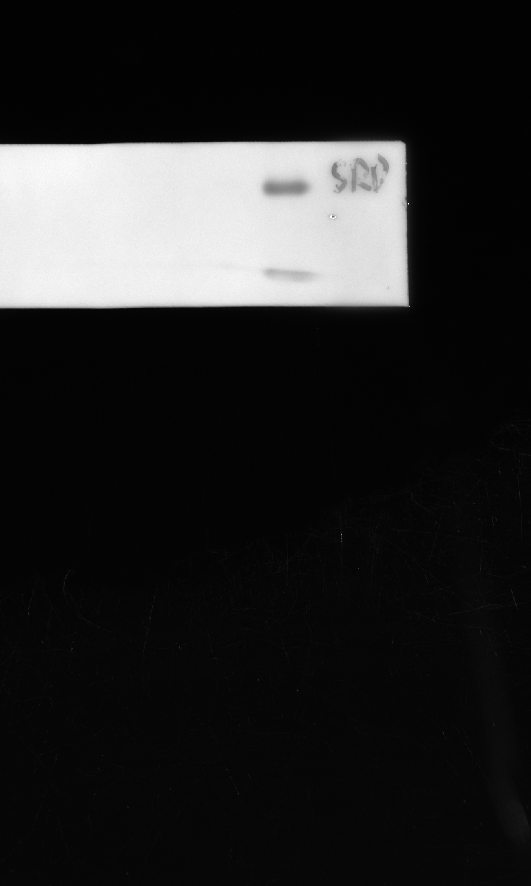

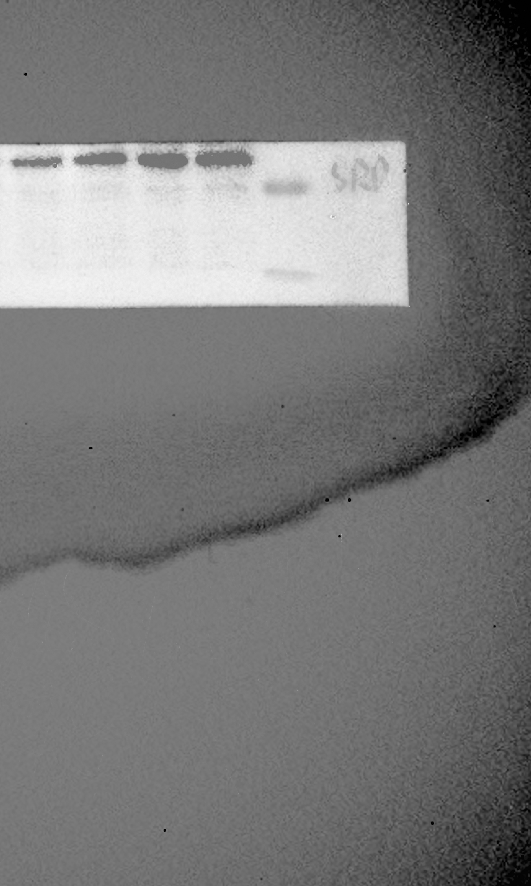

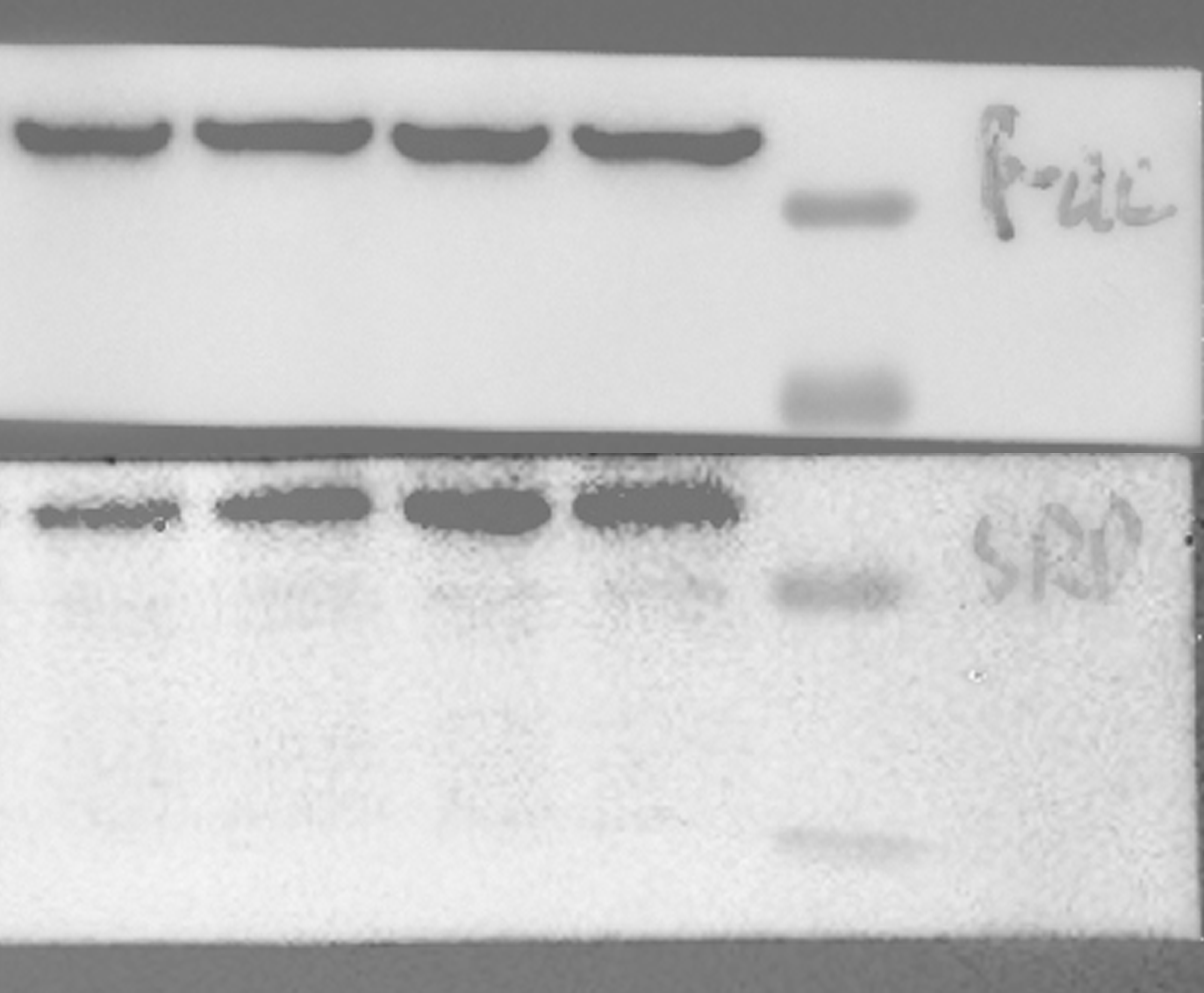


3^rd^ beta-actin_Chemiluminescence → beta-actin_brightfield → beta-actin_merge


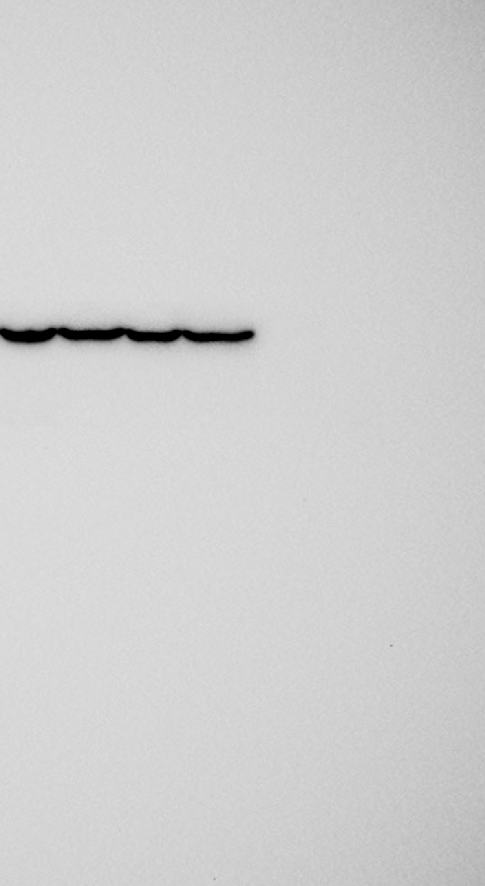

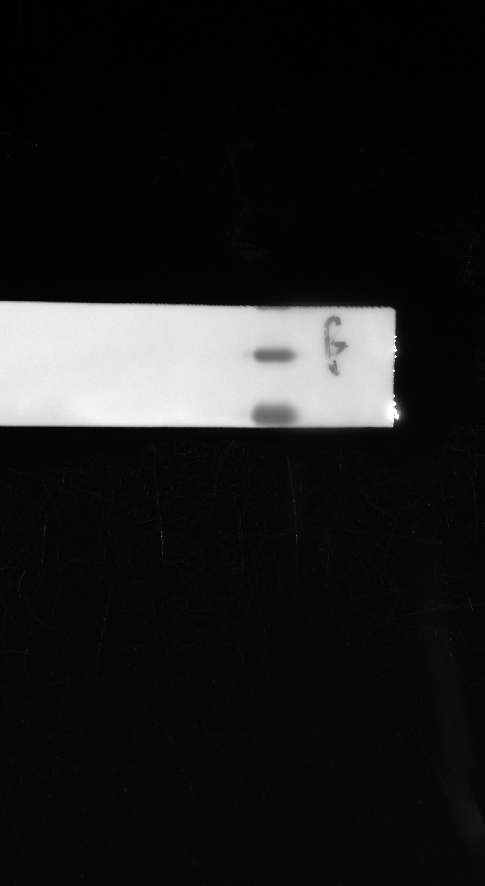

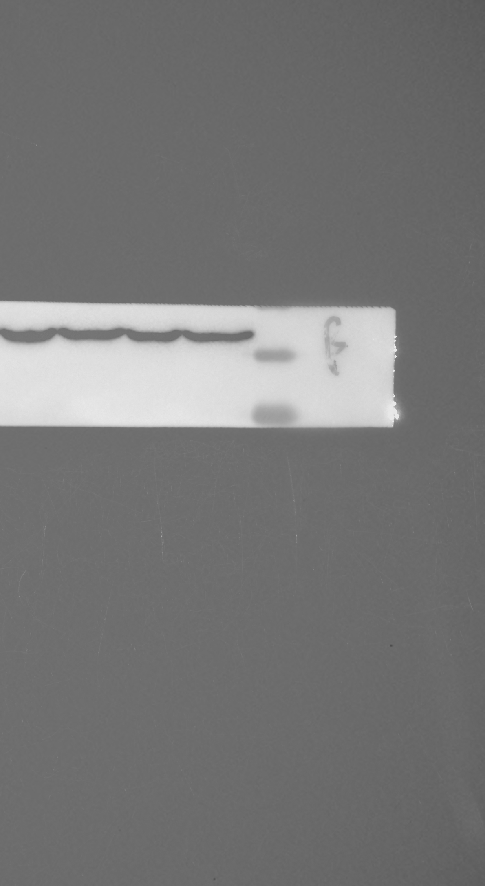


3^rd^ SRD5A2_Chemiluminescence → SRD5A2_brightfield → SRD5A2_merge → SRD5A2_beta-actin_complication


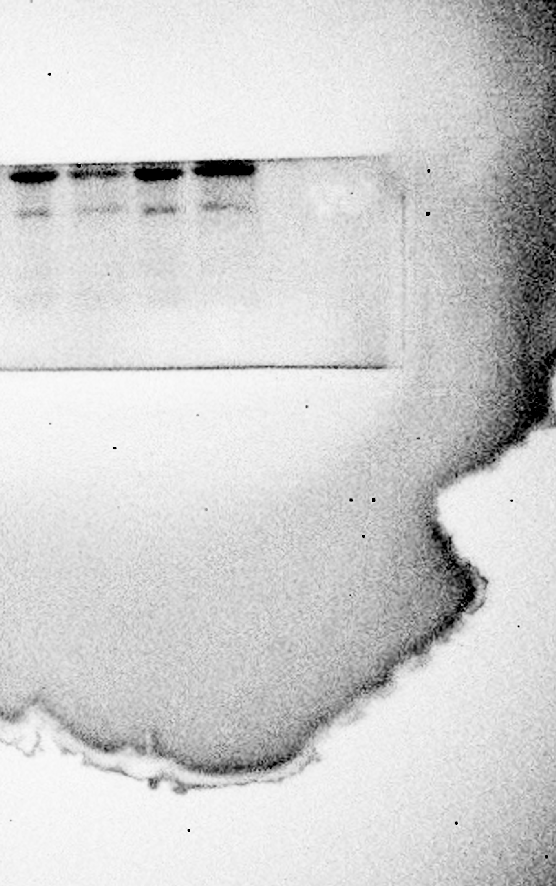

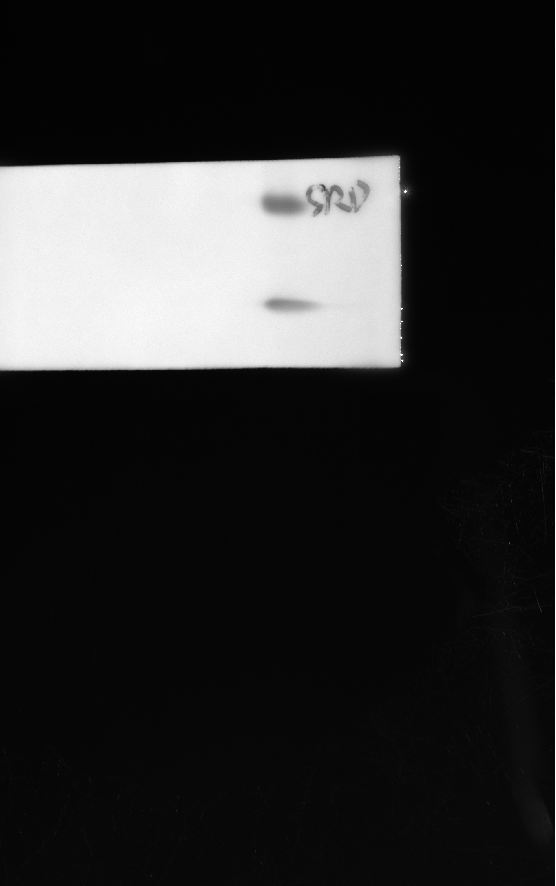

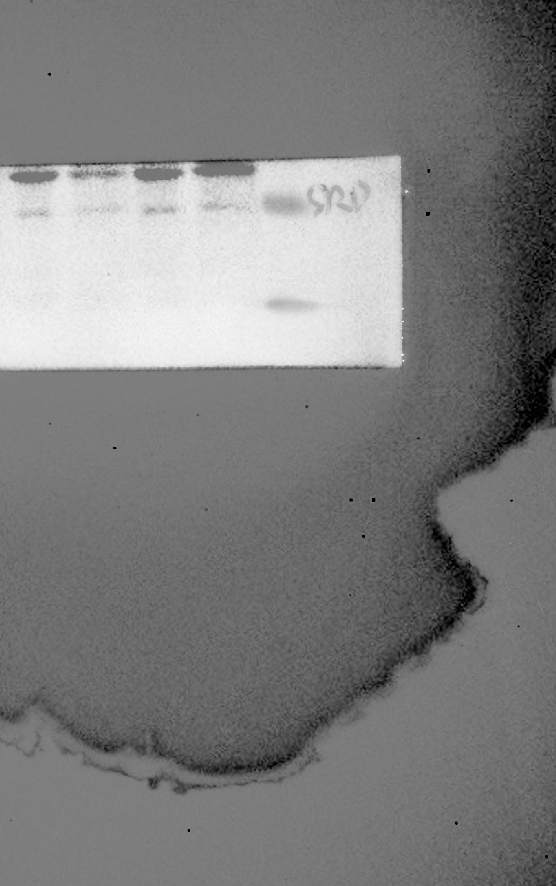

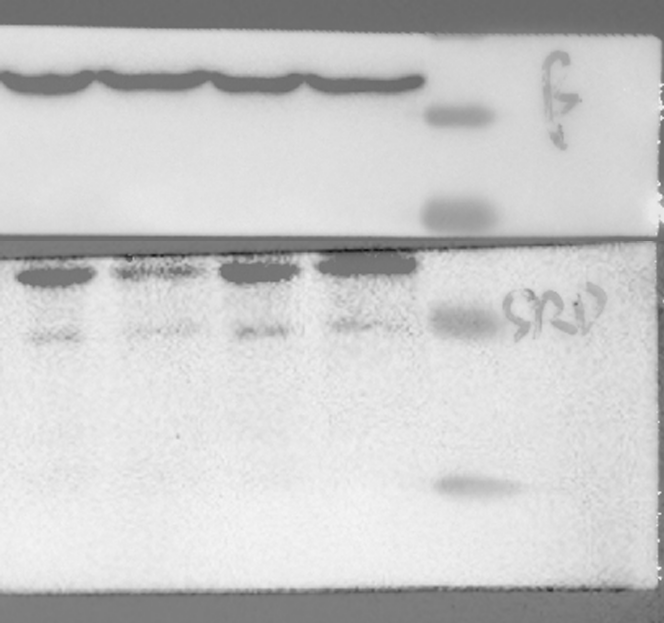

Supplement: Supplementary file 3 — Additional file 3: Figure S1. Original images of WB. [file 12906_2022_3530_MOESM3_ESM.docx]
